# Asymmetric 1,4-bis(ethynyl)bicyclo[2.2.2]octane rotators via monocarbinol functionalization. Ready access to polyrotors

**DOI:** 10.3762/bjoc.11.202

**Published:** 2015-10-09

**Authors:** Cyprien Lemouchi, Patrick Batail

**Affiliations:** 1 Laboratoire MOLTECH-Anjou, Université d’Angers, CNRS UMR 6502, 2 Boulevard Lavoisier, 49045 Angers, France

**Keywords:** bridging ligands, molecular rotors, polyrotors

## Abstract

Asymmetric rotators with a 1,4-bis(ethynyl)bicyclo[2.2.2]octane (BCO) core are needed for engineering crystalline arrays of functional molecular rotors. Their synthesis uses carbinol, 2-methyl-3-butyn-2-ol, as a protecting group because of its polar character and its ability to sustain orthogonal functionalization with the further advantage of being readily removed. The synthesis in good yields of unprecedented asymmetric rotors and polyrotors demonstrates the efficiency of this strategy.

## Findings

Synthesis-informed design [1] of rotors is essential to the development of molecular machines [2–5]. Recent advances in the chemistry of functional rotors with a 1,4-bis(ethynyl)bicyclo[2.2.2]octane (BCO) rotator core have included the design of ultra-fast rotors [6–8], the evocation of the phenomena of quantum dissipation in a hybrid system of BCO and organic conductors [9], and the discovery of a correlated motion in cogwheel pairs of BCO rotators [10], that may be switched on and off [11], in one-dimensional arrays of crystalline dirotors. Although the latter system has been created by a designed [10,12], albeit not general, synthesis based on the asymmetrical (4-pyridylethynyl)-4-ethynylbicyclo[2.2.2]octane [13], this chemistry has remained centered on symmetric precursors and targets. We disclose in this paper an efficient route that provides a general entry into asymmetric 1,4-bis(ethynyl)bicyclo[2.2.2]octane (BCO) rotators. The importance of this synthetic advance is illustrated by three examples demonstrating the ready accessibility to an even larger diversity of polyrotors.

**Scheme 1:**
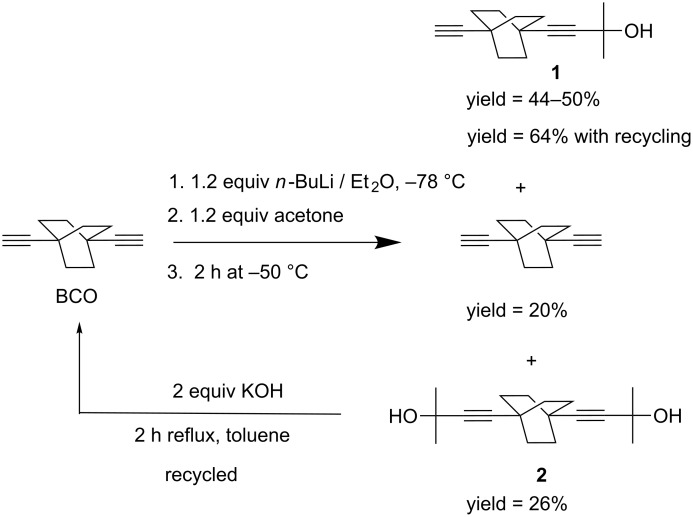
Synthesis of the asymmetric rotor **1**.

The salient feature of this synthesis lies in its generic character because 4-(4-ethynylbicyclo[2.2.2]octan-1-yl)-2-methylbut-3-yn-2-ol, (**1**) (Scheme 1), the single precursors of many diverse targets, is readily obtained. The choice of the carbinol protecting group was dictated on account of its notable polar character that makes for efficient chromatographic separation steps. In addition, the carbinol group offers large opportunities for orthogonal functionalization and it is readily removed [14–15]. As reported herein, this proved quite efficient in the development of a large variety of functionalization sequences of BCO rotators by performing nucleophilic reactions on the terminal alkyne [16] as well as Sonogashira coupling reactions [17].

One way to synthesize **1** is via a catalytic reaction to achieve the deprotection of a single 2-methyl-3-butyn-2-ol and transform the biscarbinol **2** into the asymmetrical monocarbinol derivative **1**. The procedure requires only minute amounts, that is less than 10 mol % of base, in order to prevent the full deprotection; in our hands, this yielded only small amounts of materials, as reported for other systems in the literature [18]. Therefore, we have chosen to explore the nucleophilic addition to acetone of the monometallated acetylenide, a reaction that requires a precise control of both the amount of *n*-BuLi/acetone reactants and temperature. Thus, after chromatographic separation, **1** is readily obtained with excellent purity and good yields (44–50%, Scheme 1). One notable advantage of this strategy lies in its ability to recycle the side compound **2** back to BCO, the starting material, allowing to increase the yield of the monocarbinol **1** up to 64% (Scheme 1).

**Scheme 2:**
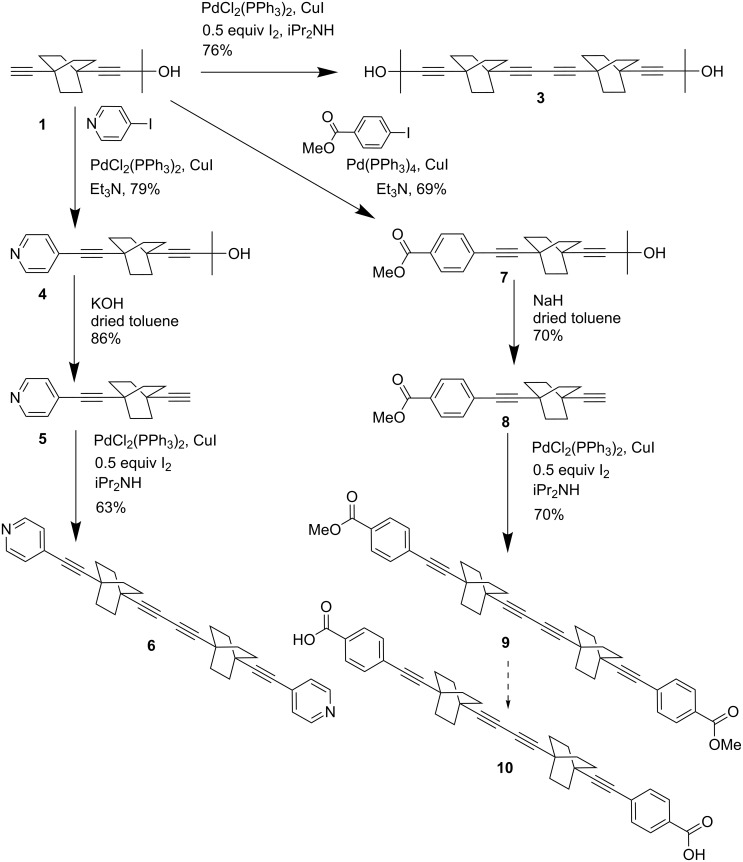
Synthesis of dirotors **6** and **10**.

The benefit of this simple approach is illustrated by the synthesis of dirotors in higher yield than our former [10,12] synthesis of **6** and with very diverse functional groups (Scheme 2). Hence, large quantities of the diyne diester dirotor, **9** are obtained (Scheme 2) providing ready, on-demand access to **10**.

The dirotors **3**, **6** and **9** are synthesized by palladium-catalyzed homocoupling [19] of the mono-terminated alkene BCOs **1**, **5** and **8**, respectively (Scheme 2). Note that the scope of our earlier synthesis was much narrower since it only allows the preparation of a monopyridine derivative, **5** subsequently engaged to yield **6** and could not be upscaled to produce the targets in gram scale. These limitations are lifted by our novel strategy. Hence, **5** is obtained in a better yield and a higher purity. Besides, this novel approach delivers the monosubstituted BCOs **1** and **8** and, in turn, the alcohol and carboxylate-functionalized dirotor diynes, **3** and **9**. It is important to note that **8** was obtained from **7** by a modified deprotection procedure of the carbinol group using NaH instead of KOH under dry conditions, thereby allowing the deprotection to proceed succesfully while preventing the ester saponification [19–20]. Indeed, keeping the ester function is of primary importance for solubility considerations, as it enhances the reactivity in the homocoupling reaction and also facilitates the purification of **9** by chromatography on silica gel. This yielded the dicarboxylate diyne dirotor **10** with the high purity required for the preparation of framework solids.

**Scheme 3:**
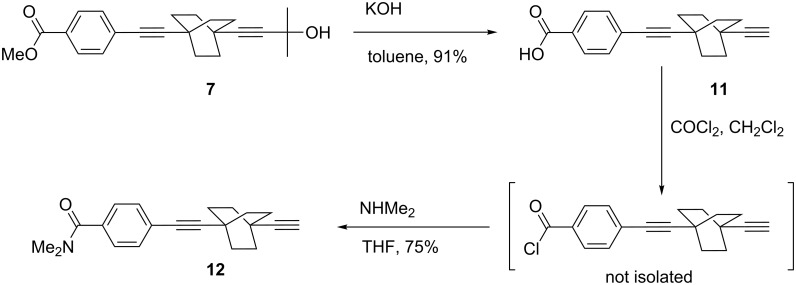
Synthesis of the tertiary amide **12**.

Saponification of the ester function and carbinol deprotection of **7** are carried out in one single step to yield to the carboxylic acid **11**. The tertiary amide **12** was then prepared in good yield and high purity by reacting the activated acyl chloride with *N*,*N*-dimethylamine (Scheme 3).

**Scheme 4:**
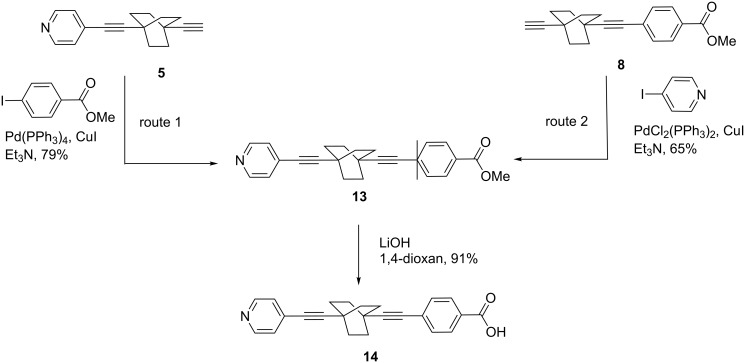
Synthesis of the extended alkaloid ligand **14**.

A further benefit of our synthesis lies in the obtainment of **14** (Scheme 4), a new class of extended alkaloid ligands similar to isonicotinic acid (of much smaller spatial extension) which was obtained recently in a synthesis of a simple Li(I) salt with an extended framework by Abrahams, Robson and co-workers [21].

Compound **13** was prepared either from **5** or **8** by Sonogashira coupling reactions (Scheme 4). Route 1 was preferred on account of higher yields. The ester function is necessary to purify the intermediate **13** and isolate **14** with excellent purity.

These results demonstrate the viability of our approach of the desymmetrization of 1,4-bis(ethynyl)bicyclo[2.2.2]octane rotators. The good yields allow the large amounts required for self-assembly and subsequent investigations of the dynamics of the rotors in the solid state to be readily prepare. The chemistry, physical chemistry and materials chemistry and physics of asymmetric rotors and their potential in the development of molecular machines that can perform mechanical functions can now be systematically studied.

## Supporting Information

File 1 Experimental section.
