# Supplementary material for: Asymmetric 1,4-bis(ethynyl)bicyclo[2.2.2]octane rotators via monocarbinol functionalization. Ready access to polyrotors
Source: Beilstein J Org Chem. 2015 Oct 9;11:1881–5. doi: 10.3762/bjoc.11.202 (PMC4660993; doi:10.3762/bjoc.11.202)
Supplement: File 1 — Experimental section. [file Beilstein_J_Org_Chem-11-1881-s001.pdf]

Supporting Information

for

Asymmetric 1,4-bis(ethynyl)bicyclo[2.2.2]octane rotators via monocarbinol functionalization. Ready access to polyrotors

Cyprien Lemouchi* and Patrick Batail*

Address: Laboratoire MOLTECH-Anjou, Université d'Angers, CNRS UMR 6502, 2 Boulevard Lavoisier, 49045 Angers, France

Email: Cyprien Lemouchi - cyprien.lemouchi@ensicaen.fr; Patrick Batail - patrick.batail@univ-angers.fr

*Corresponding author

Experimental section

Contents

Synthesis and characterization of compounds

1.1	Synthesis and characterization of 4-(4-ethynylbicyclo[2.2.2]oct-1-yl)-2-methylbut-3-yn-2-ol (**1**) (includes **2**)	s3
1.2	Synthesis and characterization of bis((4-(2-methylbut-3-yn-2-ol)-ethynyl)bicyclo[2.2.2]oct-1-yl)buta-1,3-diyne (**3**)	s6
1.3	Synthesis and characterization of 2-methyl-4-(4-(pyridin-4-ylethynyl)bicyclo[2.2.2]oct-1-yl)but-3-yn-2-ol (**4**)	s8
1.4	Synthesis and characterization of 4-((4-ethynylbicyclo[2.2.2]oct-1-yl)ethynyl)pyridine (**5**)	s11
1.5	Synthesis and characterization of bis((4-(4-pyridyl)-ethynyl)bicyclo[2.2.2]oct-1-yl)buta-1,3-diyne (**6**)	s13
1.6	Synthesis and characterization of methyl-4-((4-(3-hydroxy-3-methylbut-1-ynyl)bicyclo[2.2.2]oct-1-yl)ethynyl)benzoate (**7**)	s15
1.7	Synthesis and characterization of methyl 4-((4-ethynylbicyclo[2.2.2]oct-1-yl)ethynyl)benzoate (**8**)	s18
1.8	Synthesis and characterization of dimethyl 4,4'-(4,4'-(buta-1,3-diyne-1,4-diyl)bis(bicyclo[2.2.2]oct-4,1-diyl))bis(ethyne-2,1-diyl)dibenzoate (**9**)	s21
1.9	Synthesis and characterization of 4-((4-ethynylbicyclo[2.2.2]oct-1-yl)ethynyl)benzoic acid (**11**)	s23
1.10	Synthesis and characterization of 4-((4-ethynylbicyclo[2.2.2]oct-1-yl)ethynyl)-N,N-dimethylbenzamide (**12**)	s26
1.11	Synthesis and characterization of methyl 4-((4-(pyridin-4-ylethynyl)bicyclo[2.2.2]oct-1-yl)ethynyl)benzoate (**13**)	s29
1.12	Synthesis and characterization of 4-((4-(pyridin-4-ylethynyl)bicyclo[2.2.2]oct-1-yl)ethynyl)benzoic acid (**14**)	s32

References for Supporting Information

1. Lemouchi, C.; Iliopoulos, K.; Zorina, L.; Simonov, S.; Wzietek, P.; Cauchy, T.; Rodríguez-Fortea, A.; Canadell, E.; Kaleta, J.; Michl, J.; Gindre, D.; Chrysos, M.; Batail, P. *J. Am. Chem. Soc.* **2013**, *135*, 9366;
2. Lemouchi, C *Doctoral Thesis*, Université d'Angers, 2010
3. Lemouchi, C.; Barrès, A.-L.; Mézière, C.; Rondeau, D.; Zorina, L.; Wzietek, P.; Batail, P. *Dalton Trans.* **2011**, *40*, 8075.
4. Havens, J. S. *J. Org. Chem.* **1984**, *50*, 1763.

1.1 Synthesis and characterization of 4-(4-ethynylbicyclo[2.2.2]oct-1-yl)-2-methylbut-3-yn-2-ol (1)

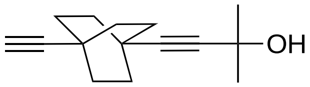

To a solution of 1,4-diethynylbicyclo[2,2,2]octane (1 g, 6.32 mmol) in diethyl ether (70 mL) under argon, *n*-butyllithium (3 mL of 2.5 M soln. in hexane, 7.50 mmol) was slowly added at −78 °C. The reaction mixture was stirred at −78 °C for 30 min, then acetone (560 µL, 7.60 mmol) was added dropwise at −78 °C. Afterwards, the reaction mixture was slowly warmed up to −50 °C, then stirred for 2 h while TLC-monitored (eluent: ethyl acetate/*n*-heptane 1:1). Finally, the reaction was quenched with an aqueous saturated ammonium chloride solution. The mixture was extracted with dichloromethane and dried over $MgSO_4$. The crude material was purified by silica gel column chromatography using 100% *n*-heptane then ethyl acetate/*n*-heptane (15:85 to 50:50) in successive gradients. The first elute was the starting material 1,4-diethynylbicyclo[2,2,2]octane (250 mg, 1.58 mmol), then the asymmetrical BCO, **1** (600 mg, 2,78 mmol) as a white powder with 44% isolated yield (brought up to 64% yield by recycling) mp: 121 °C ; ^{1}H NMR ($CDCl_3$, 300 MHz) δ 2.0 (s, 1H), 1.8 ($s_{(broad)}$, 1H), 1.7 (m, 12H), 1.4 (s, 6H); ^{13}C NMR ($CDCl_3$, 300 MHz) δ 91.38, 88.76, 85.39, 68.29, 65.36, 32.01, 31.91, 31.88, 26.23, 26.11; Anal. Calcd for $C_{15}H_{20}O$ C, 83.28 ; H, 9.32. Found: C, 82.61; H, 9.40. Finally, the symmetrical-BCO, 4,4'-(bicyclo[2.2.2]oct-1,4-diyl)bis(2-methylbut-3-yn-2-ol) (**2**, 450 mg, 1,64 mmol) was isolated as a white powder with 26 % yield. mp: 114.7 °C; ^{1}H NMR ($CDCl_3$, 300 MHz) δ 1.9 (sl, 2H), 1.7 (s, 12H), 1.4 (s, 12H); ^{13}C NMR ($CDCl_3$, 300 MHz) 88.97, 85.28, 65.40, 32.03-32.0, 26.13; anal. calcd for $C_{18}H_{26}O_2$ C, 78.79; H, 9.55, found: C, 77.53; H, 10.08. After a recycling by column chromatography, 20% of the unreacted starting material, the 1,4-diethynylbicyclo[2.2.2]octane, was recovered.

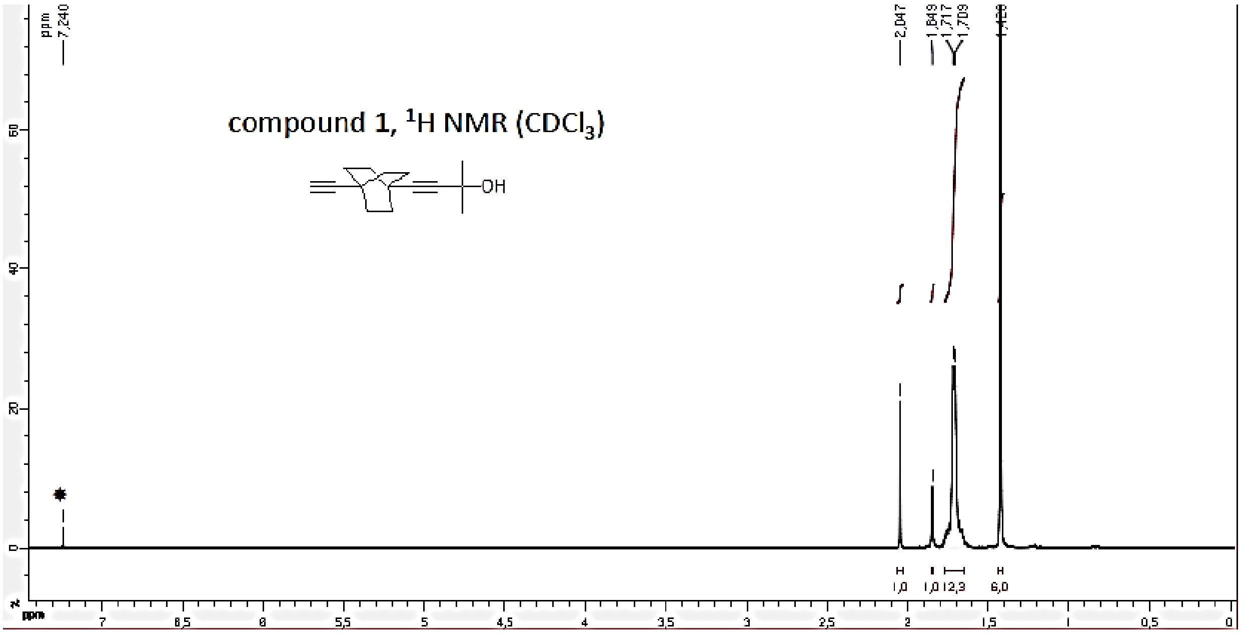

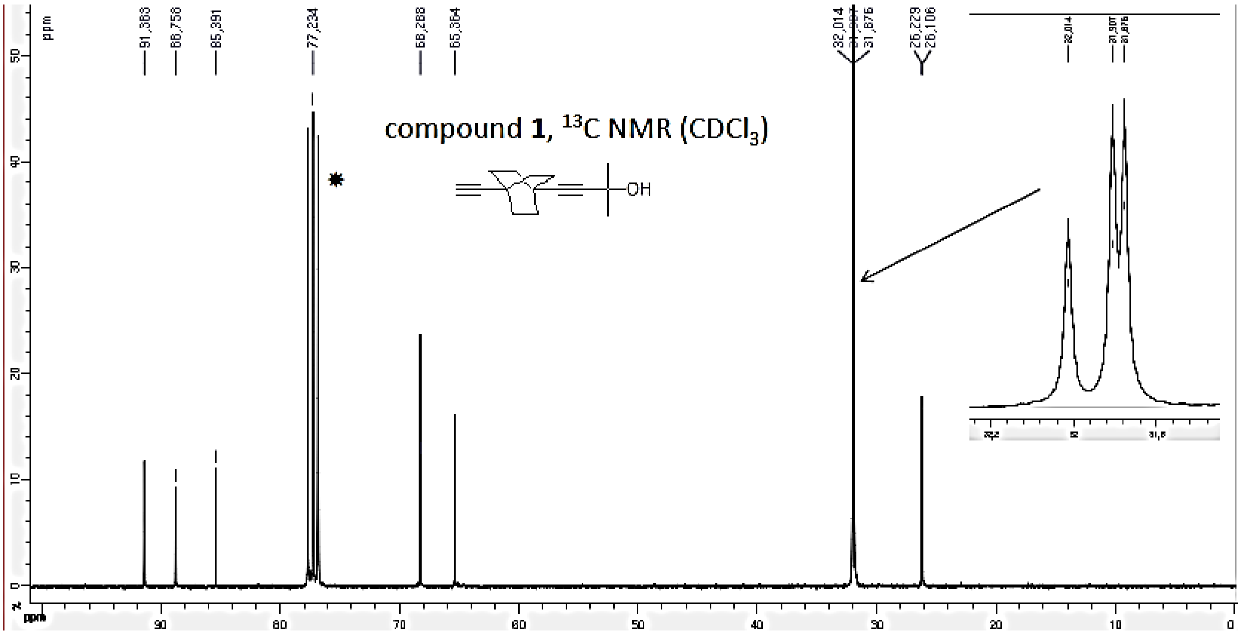

Figure S1. ^{1}H and ^{13}C NMR of compound **1** at 300 MHz. On each spectrum the solvent peak is labeled with a star.

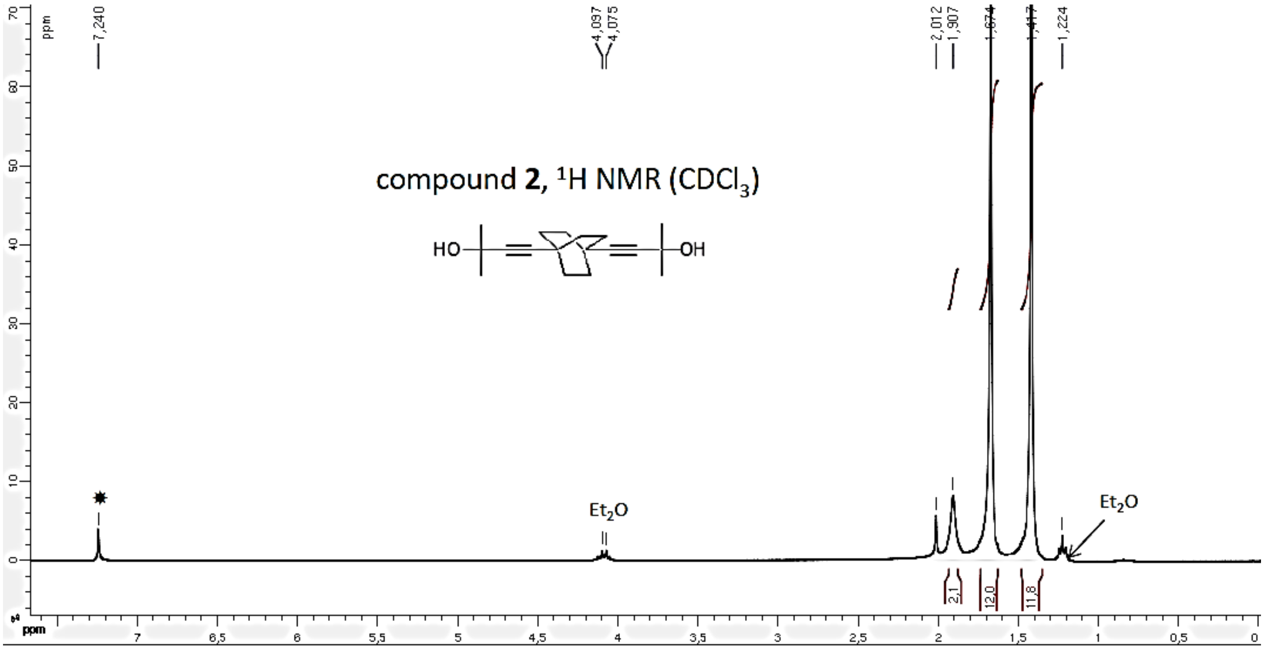

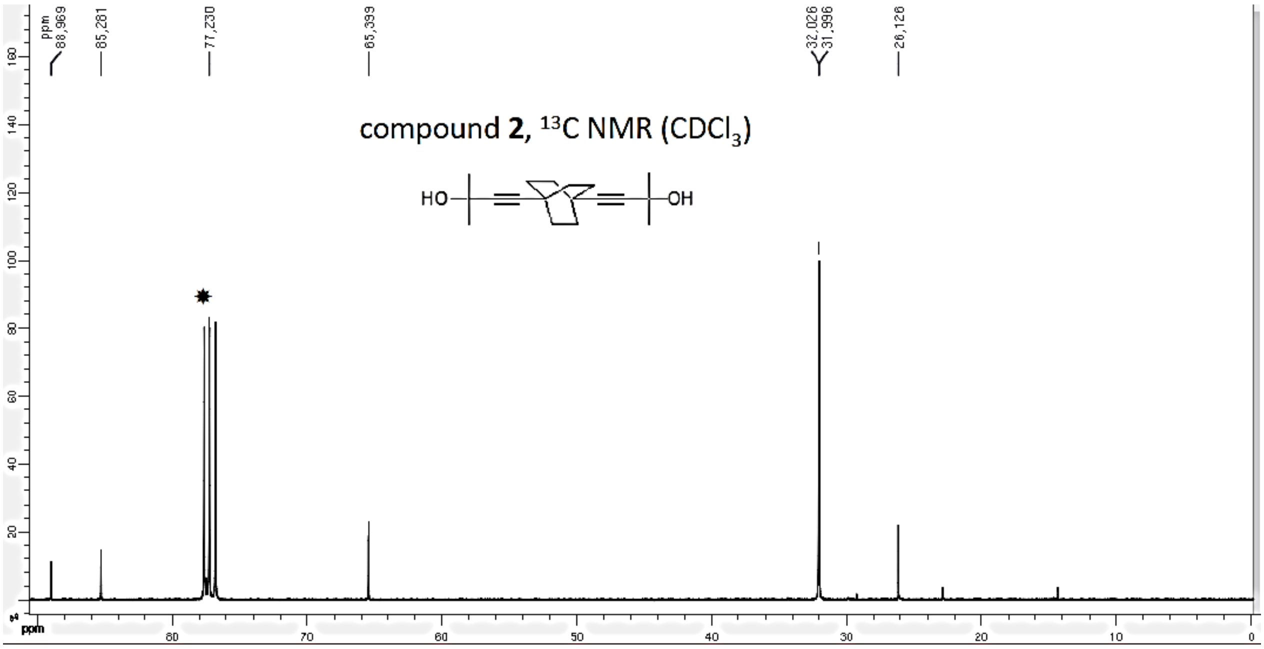

Figure S2. ^{1}H and ^{13}C NMR of compound **2** at 300 MHz. On each spectrum the solvent peak is labeled with a star.

1.2 Synthesis and characterization of 1,4-bis((2-methylbut-3-yn-2-ol)bicyclo[2.2.2]oct-1,4-diyl))buta-1,3-diyne (3)

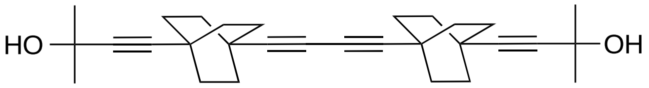

To a solution of 4-(4-ethynylbicyclo[2.2.2]oct-1-yl)-2-methylbut-3-yn-2-ol (**1**), (300 mg, 1,39 mmol) in *N*,*N*-diisopropylamine (200 mL) sparged with argon for 15 min, catalytic amounts (ca. 14 mol %) of [$PdCl_2(PPh_3)_2$] and CuI (15 mol %) and sublimated iodine (177 mg, 0.69 mmol) were added. The reaction mixture was stirred at room temperature for 2 h under argon. The reaction progress was monitored by thin layer chromatography (ethyl acetate/*n*-heptane 50:50). The reaction mixture was cooled down, diluted with methylene chloride and washed with ammonium chloride saturated solution. The organic layer was extracted and dried over magnesium sulfate, filtrated and the solvent was removed under vacuum. The product was purified by column chromatography with 100% *n*-heptane, ethyl acetate/*n*-heptane 10:90 and ethyl acetate/*n*-heptane 50:50 to afford 227 mg (76% isolated yield) of **3** as a white powder, which was crystallized in hot ethyl acetate to deliver colorless diamond-shaped crystals. ATG/DSC mp: 275 °C; ^{1}H NMR ($CDCl_3$, 300 MHz) δ 1.7 (sl, 24H), 1.4 (s, 12H) ; ^{13}C NMR ($CDCl_3$, 300 MHz) 88.65, 85.44, 84.69, 65.39, 65.33, 60.62, 32.01, 31.81, 31.61, 27.00, 26.14, 26.07; anal. calcd for {$[C_{30}H_{38}O_2]_2$+$[CH_3CO_2Et]$} C, 80.97; H, 8.92, found: C, 80.07; H, 8.63.

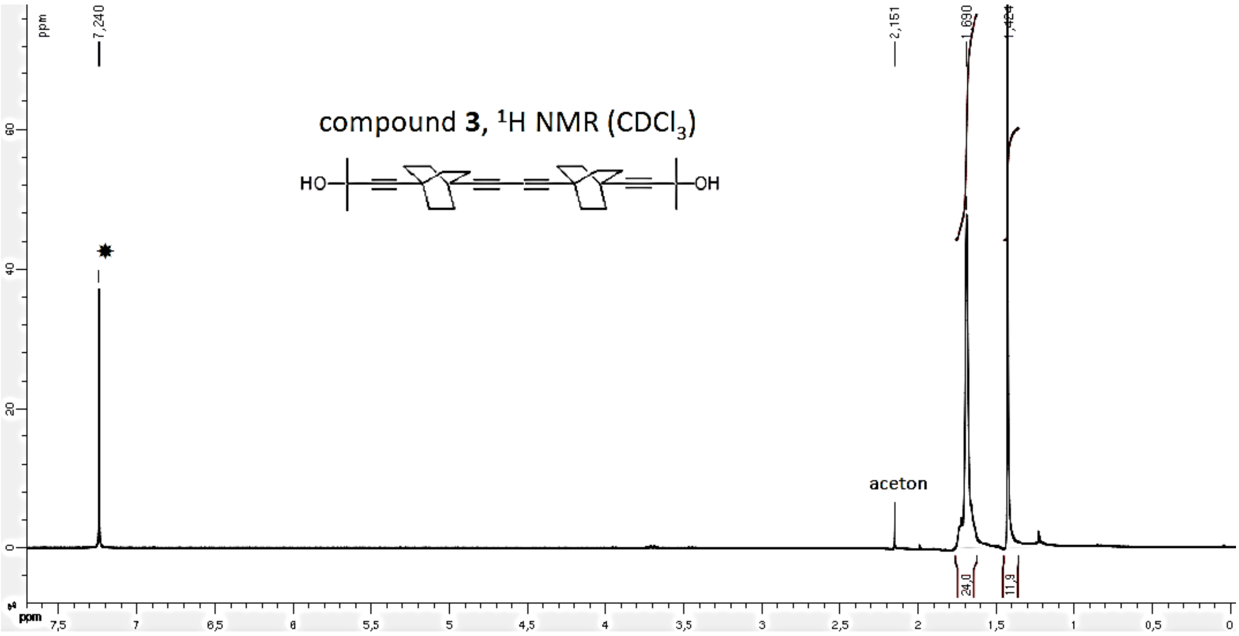

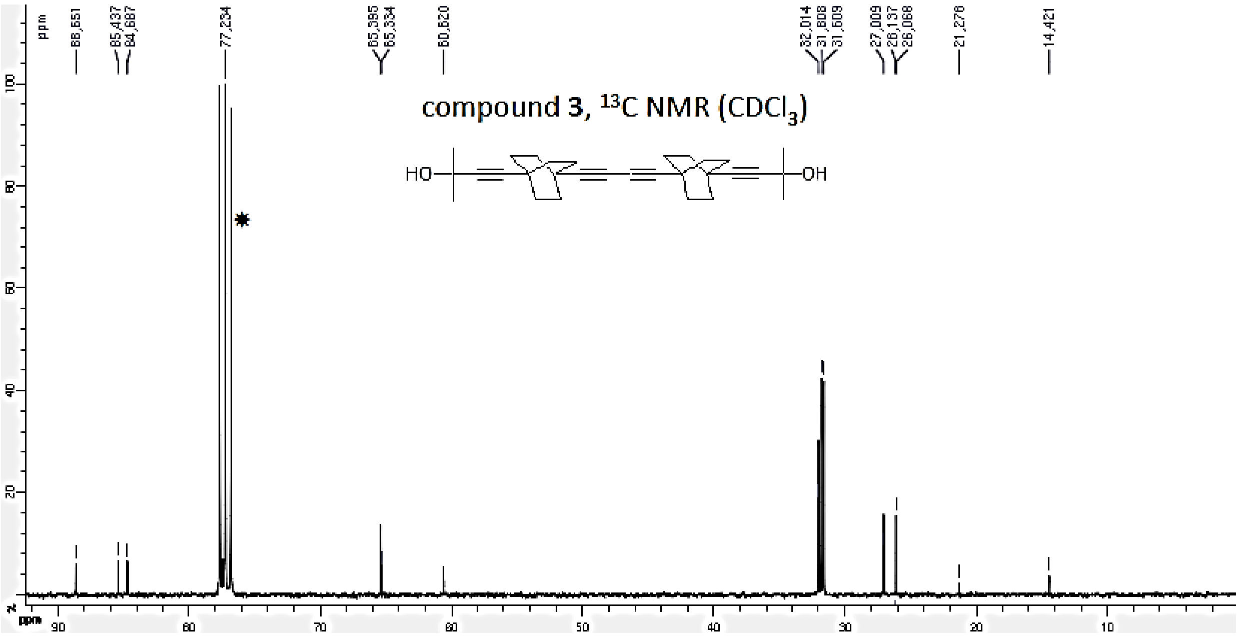

Figure S3. ^{1}H and ^{13}C NMR of compound **3** at 300 MHz. On each spectrum the solvent peak is labeled with a star.

1.3 Synthesis and characterization of 2-methyl-4-(4-(pyridin-4-ylethynyl)bicyclo[2.2.2]oct-1-yl)but-3-yn-2-ol (4)

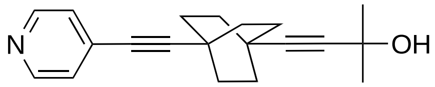

To a solid mixture of 4-iodopyridine (285 mg, 1.39 mmol), catalytic amounts (ca. 16 mol %) of [$PdCl_2(PPh_3)_2$] and CuI (15 mol %), a solution of 4-(4-ethynyl)bicyclo[2.2.2]oct-1-yl)-2-methylbut-3-yn-2-ol (**1**) (200 mg, 0.93 mmol) dissolved in triethylamine (150 mL) was sparged with argon for 15 min and finally added. The reaction mixture was heated at 60 °C overnight under argon. The reaction progress was monitored by thin layer chromatography (ethyl acetate/*n*-heptane 50:50). The reaction mixture was cooled down, poured into a mixture of ammonium chloride saturated aqueous solution and methylene chloride (50 mL/50mL) and then extracted. The organics were dried over magnesium sulfate, filtrated and the solvent was removed under vacuum. The product was purified by column chromatography (ethyl acetate/*n*-heptane 50:50) to afford 215 mg (79% isolated yield) of **4** as a yellowish crystalline powder, which was crystallized in hot ethyl acetate to afford **3** as yellowish prism-shaped crystals. mp : 180 °C ; ^{1}H NMR ($CDCl_3$, 300 MHz) δ 8.5 (d, *J* = 6 Hz, 2H), 7.2 (d, *J* = 6 Hz, 2H), 1.8 (m, 12H), 1.4 (s, 6H); ^{13}C NMR ($CDCl_3$, 300 MHz) δ 149.28, 132.79, 126.11, 102.44, 88.50, 85.61, 78.67, 65.28, 32.04, 31.88, 31.69, 27.16, 26.16; ESI(+) m/z = 294.31 [$M+H^+$]; anal. calcd for $C_{20}H_{23}NO$ C, 81.87 ; H, 7.90 ; N, 4.77 ; O, 5.45, found: C, 81.16 ; H, 7.89 ; N, 4.71 ; O, 5.42.

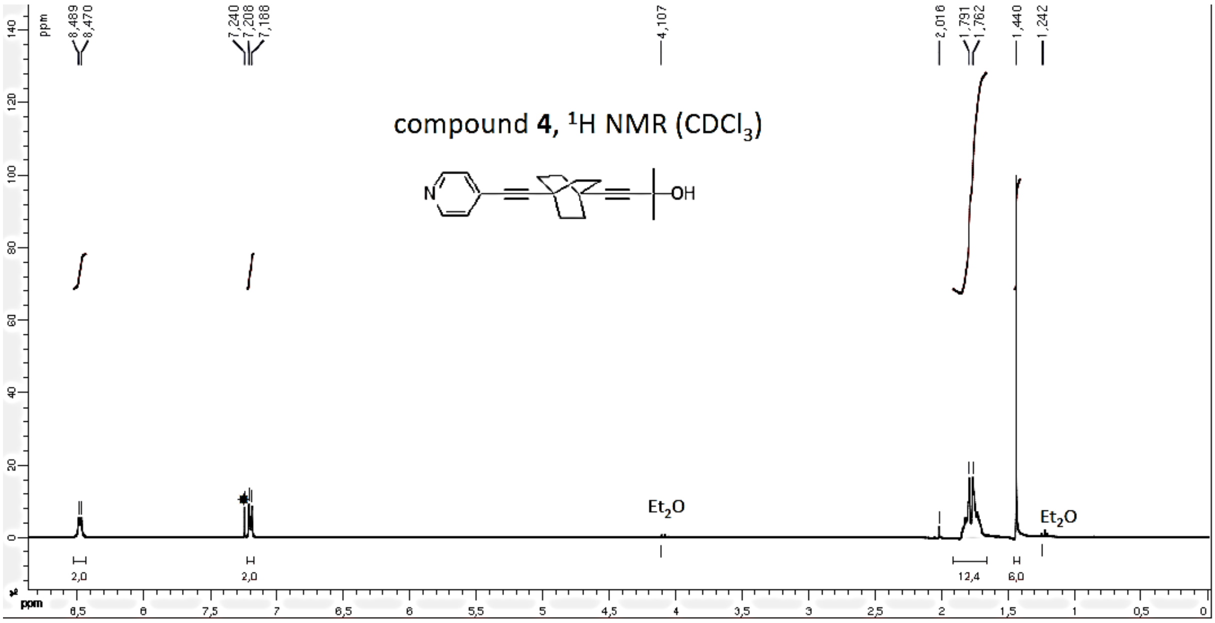

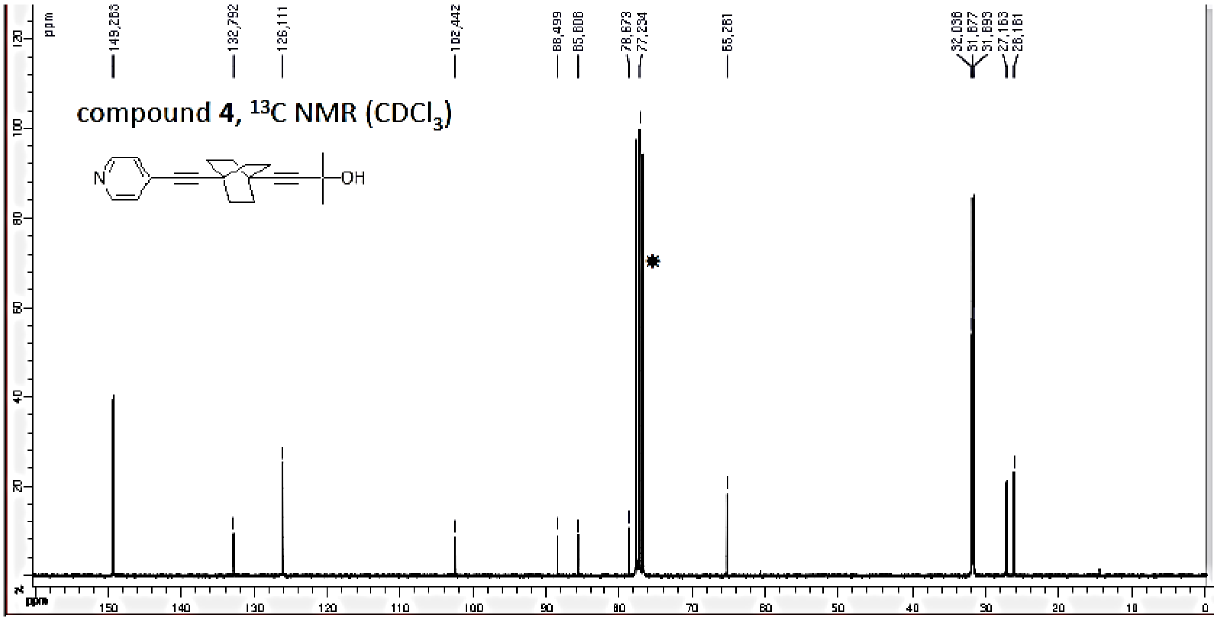

Figure S4. ^{1}H and ^{13}C NMR of compound **4** at 300 MHz. On each spectrum the solvent peak is labeled with a star.

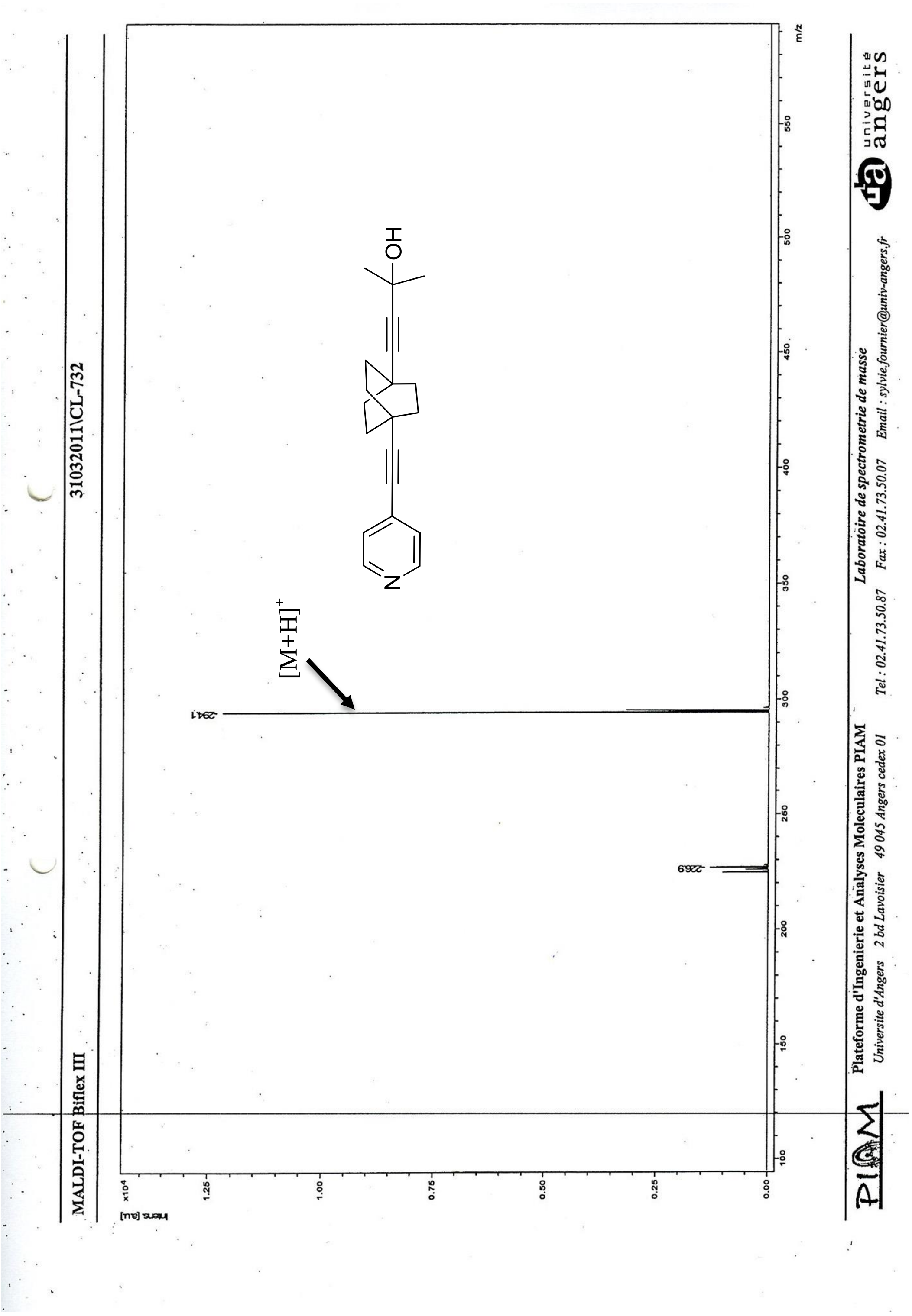

Figure S5. Mass spectra of compound 4

1.4 Synthesis and characterization of 4-((4-ethynylbicyclo[2.2.2]oct-1-yl)ethynyl)pyridine (5)

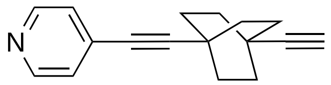

The following preparation of compound **5** is different than that previously described in Cyprien Lemouchi's doctoral thesis [2] and reference [3], yet the characterization is identical.

KOH (50 mg, 0.89 mmol) was added to a solution of 2-methyl-4-(4-(pyridin-4-ylethynyl)bicyclo[2.2.2]oct-1-yl)but-3-yn-2-ol (**4**) (260 mg, 0.89 mmol) in dry toluene (200 mL) under argon atmosphere. The mixture was heated at reflux for 2 h and the reaction monitored by TLC (ethyl acetate/*n*-heptane 50:50). The reaction mixture was filtered; the filtrate was washed with ammonium chloride saturated aqueous solution and then water. The organics were dried over magnesium sulfate, filtrated and the solvent was removed under vacuum to afford **5** (180 mg, 0.77 mmol) as a white crystalline powder in 86 % yield. For characterization data, see [2,3].

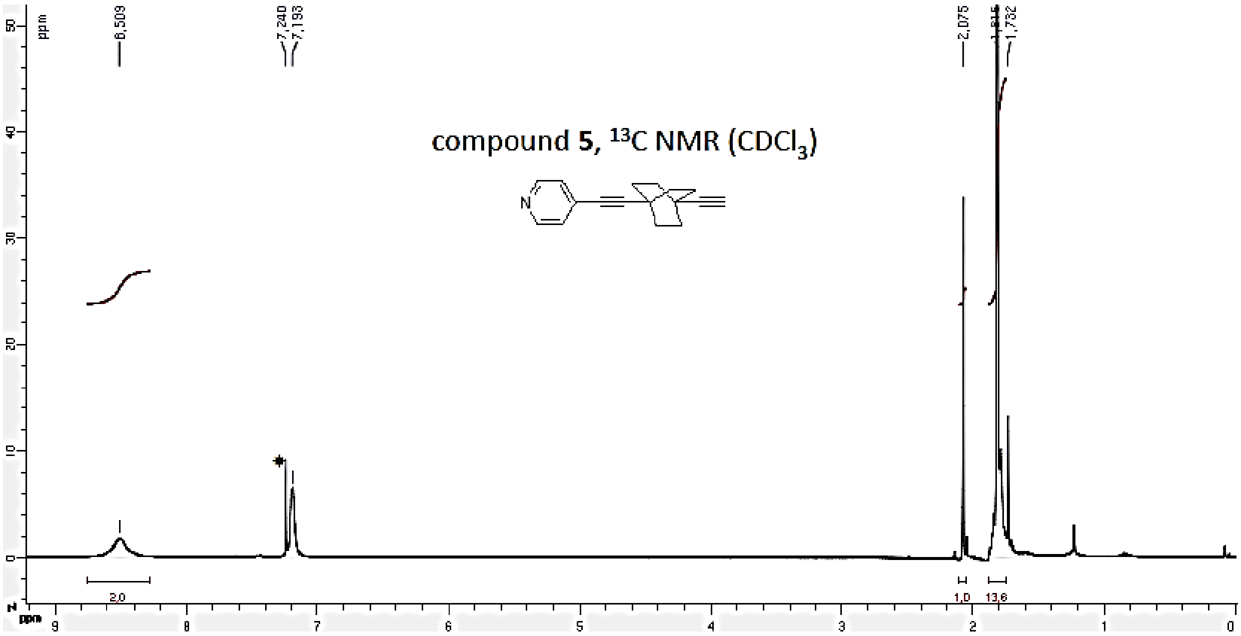

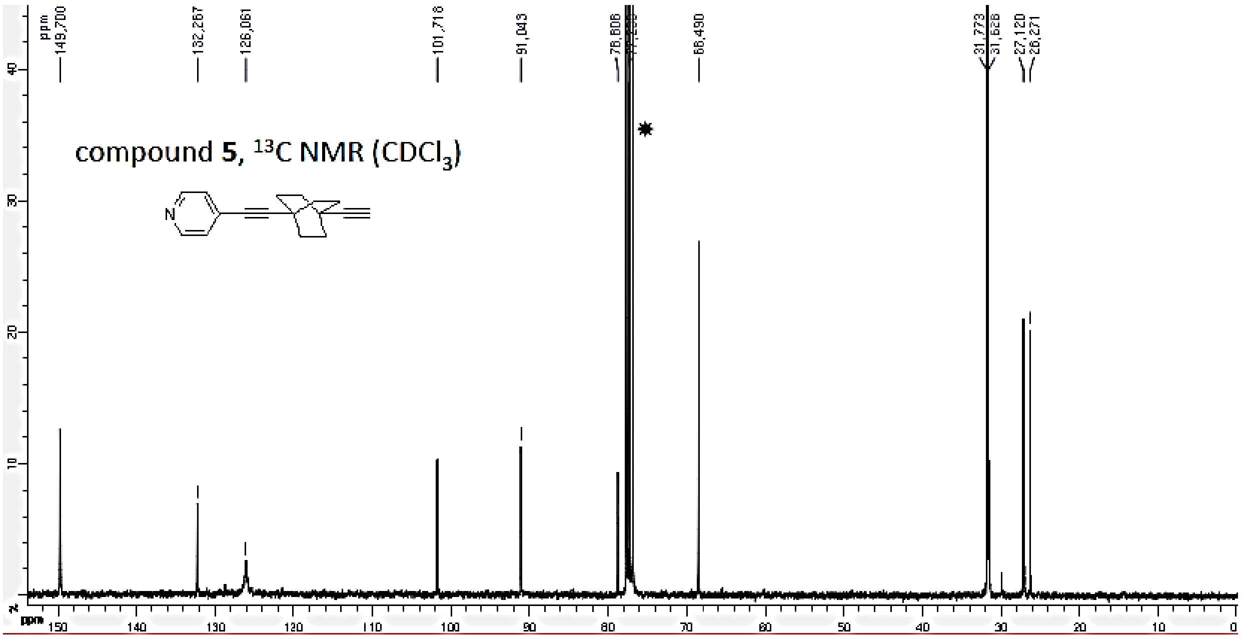

Figure S6. ^{1}H and ^{13}C NMR of compound **5** at 300 MHz. On each spectrum the solvent peak is labeled with a star.

1.5 Synthesis and characterization of bis((4-(4-pyridyl)-ethynyl)bicyclo[2.2.2]oct-1-yl)buta-1,3-diyne (6)

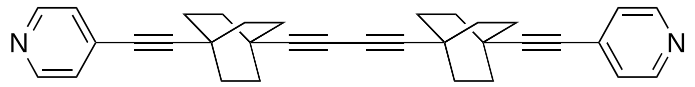

The following preparation procedure was already published in [1,2]. To a solution of 1-(4-pyridylethynyl)-4-ethynylbicyclo[2.2.2]octane (**5**) (40 mg, 0.17 mmol) in *N,N*diisopropylamin (30 mL) under argon, catalytic amounts (ca. 15 mol %) of [$PdCl_2(PPh_3)_2$] and CuI (13 mol %) were added and the mixture was degassed with argon before adding the iodine (22 mg, 0.096 mmol). The reaction mixture was stirred at room temperature for 2 h. Reaction progress was monitored by thin layer chromatography (methylene chloride/ethanol – 9:1). The reaction mixture was poured in a saturated aqueous ammonium chloride and methylene chloride (50 mL/50 mL) mixture. Organics were extracted and washed with water before being dried over magnesium sulfate, filtered and the solvent removed under vacuum. Column chromatography (methylene chloride/ethanol – 9:1) on silica gel afforded 25 mg (63% isolated yield) of **6** as a white crystalline solid. For characterization data, see [1,2].

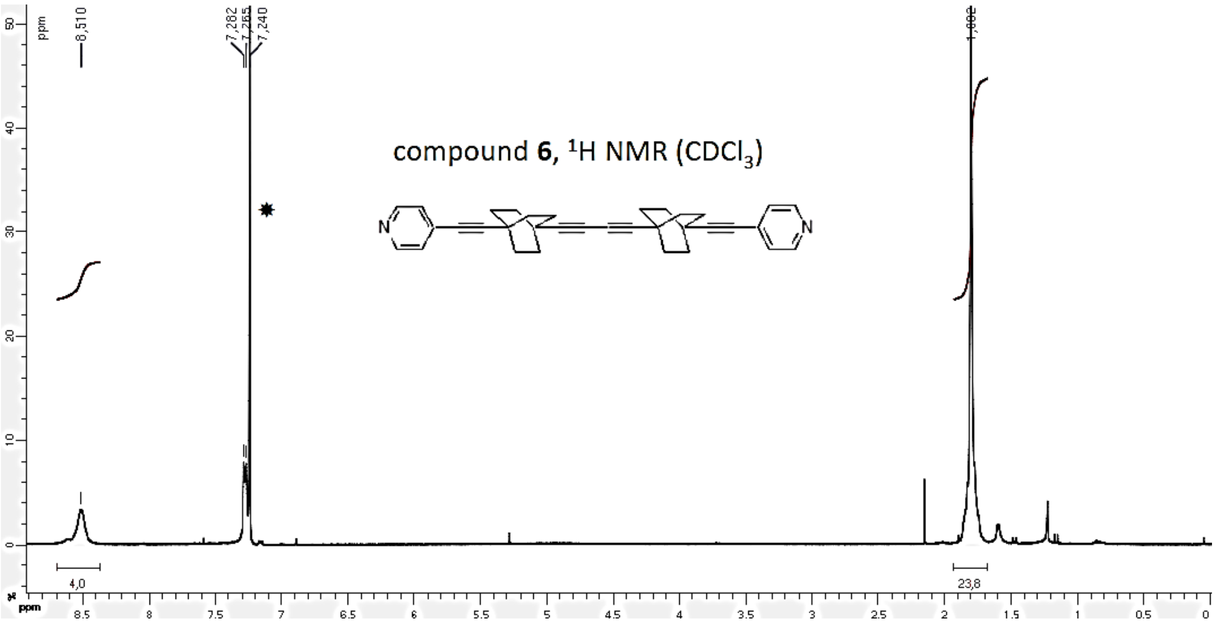

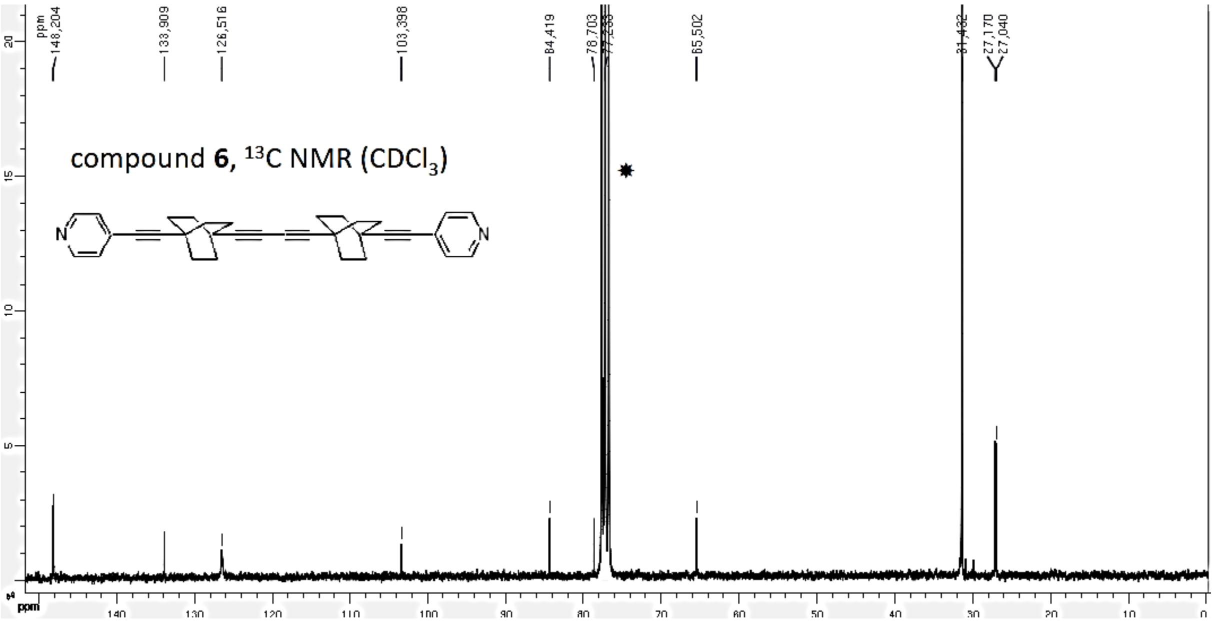

Figure S7. ^{1}H and ^{13}C NMR of compound **6** at 300 MHz. On each spectrum the solvent peak is labeled with a star.

1.6 Synthesis and characterization of methyl-4-((4-(3-hydroxy-3-methylbut-1-ynyl)bicyclo[2.2.2]oct-1-yl)ethynyl)benzoate (7)

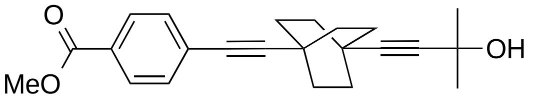

To a solid mixture of methyl 4-iodobenzoate (606 mg, 2,31 mmol), catalytic amounts (ca. 16 mol %) of [Pd(PPh_3)$_4$] and CuI (15 mol %), a solution of 4-(4-ethynylbicyclo[2.2.2]oct-1-yl)-2-methylbut-3-yn-2-ol (**1**) (250 mg, 1,16 mmol) dissolved in triethylamine (150 mL) was degassed with argon for 15 min and finally added. The reaction mixture was heated at 70 °C overnight under argon. The reaction progress was monitored by thin layer chromatography (toluene). The reaction mixture was cooled down, poured into a mixture of ammonium chloride saturated aqueous solution and methylene chloride (50 mL/50mL) and then extracted. The organics were dried over magnesium sulfate, filtrated and the solvent was removed under vacuum. The product was purified by column chromatography with toluene to afford 284 mg (69% isolated yield) of **7** as a white powder. mp: 165 °C ; ^{1}H NMR ($CDCl_3$, 300 MHz) δ 7.9 (d, *J* = 9 Hz, 2H), 7.4 (d, *J* = 9 Hz, 2H), 3.9 (s, 3H), 1.8 (m, 12H), 1.4 (s, 6H); ^{13}C NMR ($CDCl_3$, 300 MHz) δ 166.86, 131.69, 129.53, 129.03, 128.90, 100.31, 88.80, 85.45, 80.40, 65.40, 52.35, 32.04, 31.98, 31.87, 27.12, 26.20; ESI(+) m/z = 373.25 [M+Na$^+$]; anal. calcd for $C_{23}H_{26}O_3$ C, 78.83 ; H, 7.48 ; O, 13.70, found: C, 78.70 ; H, 7.48 ; O, 13.72.

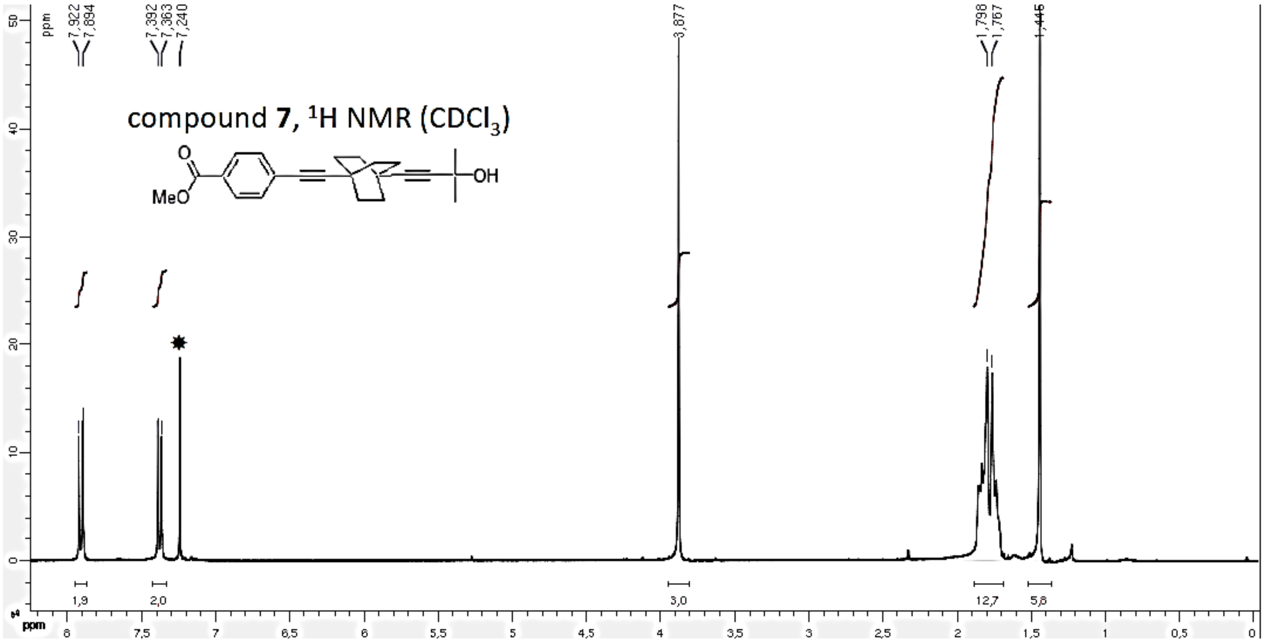

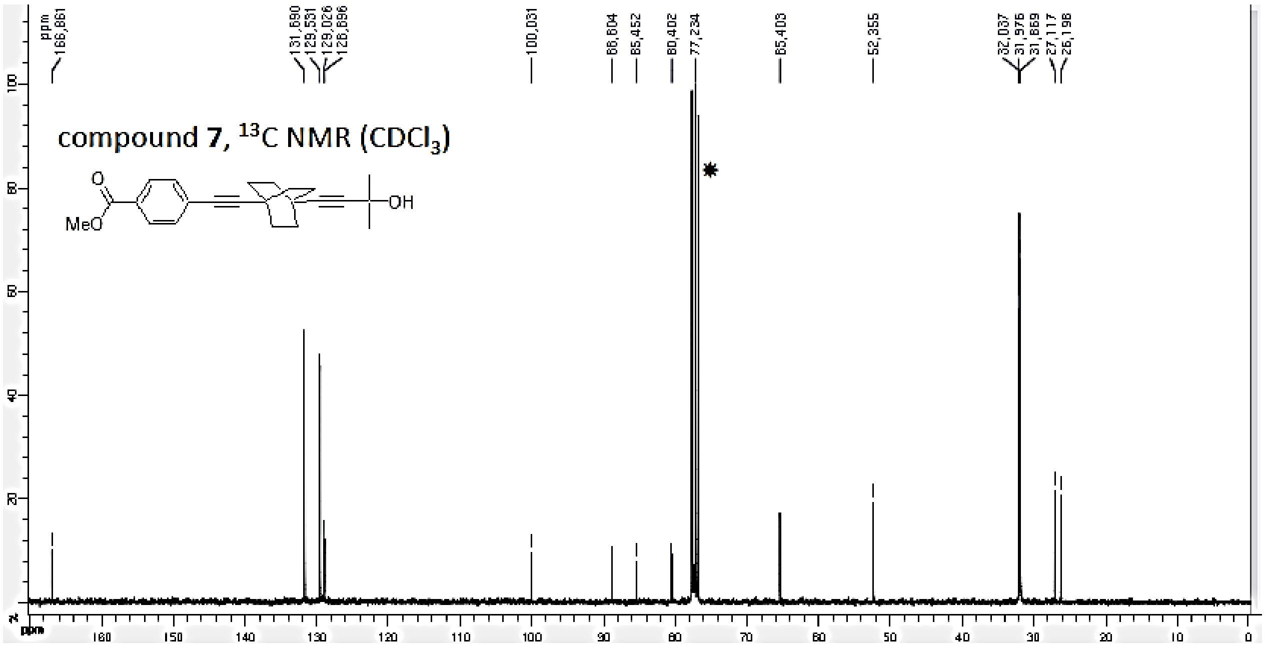

Figure S8. ^{1}H and ^{13}C NMR of compound **7** at 300 MHz. On each spectrum the solvent peak is labeled with a star.

Bruker Daltonics DataAnalysis 3.3 Page 1 of 1

Display analysis

Sample Name ***CL745***

**Analysis Info**	Acquisition Date 4/8/2011 3:05:35 PM	Operator	Ingrid
		Instrument	esquire3000 plus
Analysis Name	D:\Data\Ingrid\08042011-CL-745.d		
Method	tune esi.m		
Sample Name	CL745		
Comment			

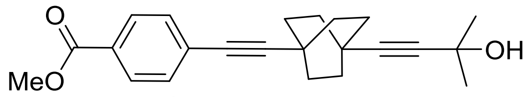

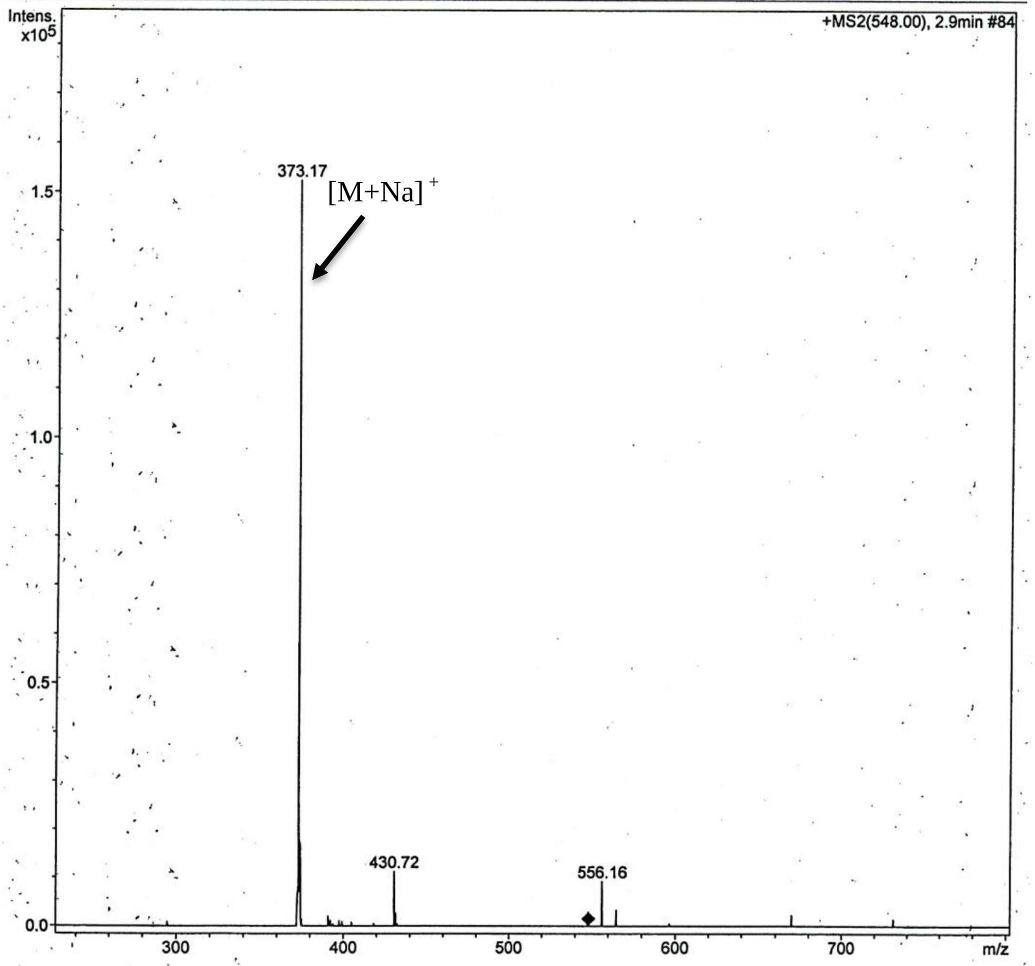

Plateforme d'Ingenierie et Analyses Moleculaires PIAM

Universite d'Angers 2 bd Lavoisier 49 045 Angers cedex 01

Laboratoire de spectrometrie de masse

Tel : 02.41.73.50.87 Fax : 02.41.73.50.07 Email : sylvie.fournier@univ-angers.fr

université angers

Figure S9. Mass spectra of compound **7**

1.7 Synthesis and characterization of methyl 4-((4-ethynylbicyclo[2.2.2]oct-1-yl)ethynyl)benzoate (8)

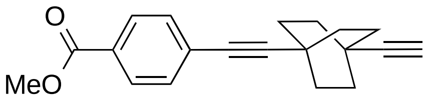

Following a published procedure [4], under rigorous anhydrous conditions, sodium hydride (60% dispersion in oil, 3 mg, 0.07 mmol) were added to a clear solution of methyl-4-((4-(3-hydroxy-3-methylbut-1-ynyl)bicyclo[2.2.2]oct-1-yl)ethynyl)benzoate (**7**) (200 mg, 0.71 mmol) in dry toluene (70 mL). The mixture was heated at reflux for 1 h. Reaction progress was monitored by thin layer chromatography, (ethyl acetate/toluene = 10:90). The white precipitate that appears upon cooling the mixture down to room temperature is filtered. The filtrate was extracted with ethyl acetate, washed with an ammonium chloride saturated solution, brine and water to yield a whitish solid residue. The product was purified by column chromatography (ethyl acetate/toluene 10:90) to afford 292 mg (70% isolated yield) of **8** as a white solid. mp : 172 °C, ^{1}H NMR ($CDCl_3$, 300 MHz) δ 7.9 (d, J = 9 Hz, 2H), 7.4 (d, J = 9 Hz, 2H), 3.9 (s, 3H), 2.1 (s, 1H), 1.8 ($s_{(broad)}$, 12H) ; ^{13}C NMR ($CDCl_3$, 300 MHz) 166.85, 131.70, 129.55, 129.08, 128.85, 99.82, 91.22, 80.48, 68.41, 52.36, 31.88, 31.80, 27.11, 26.30 ; ESI(+) m/z = 293.2 $[M+H]^+$, 315.2 $[M+Na]^+$, 606.8 $[2M+Na]^+$; anal. calcd for $C_{20}H_{20}O_2$ C, 82.16; H, 6.89, found: C, 81.09; H, 6.94.

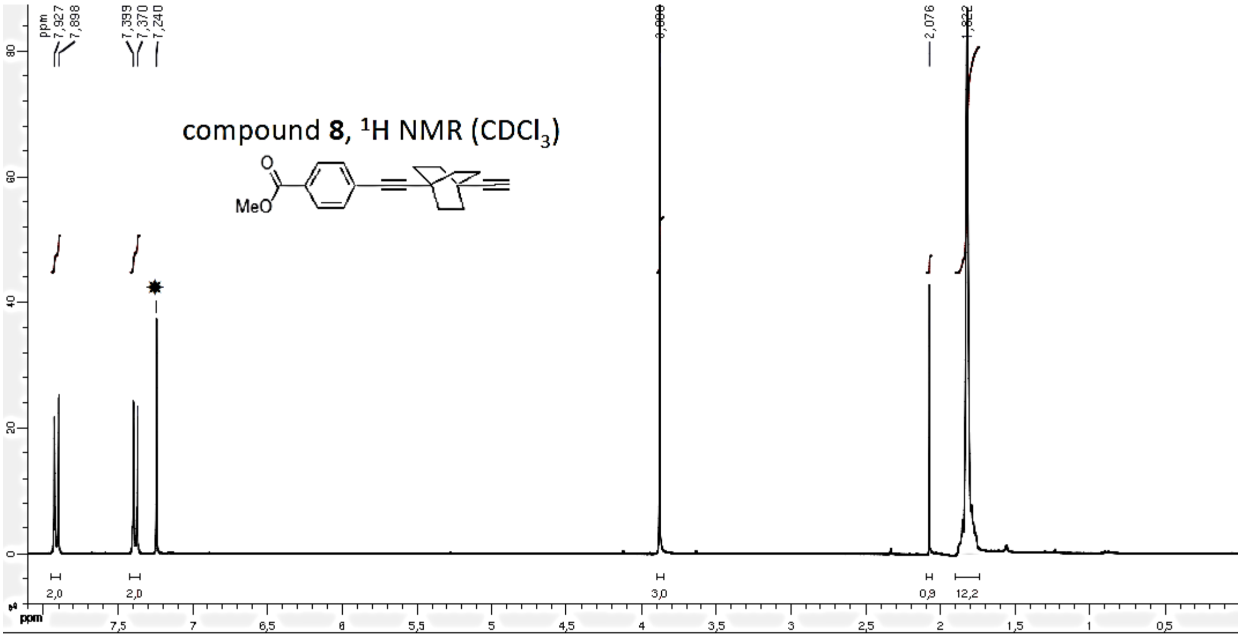

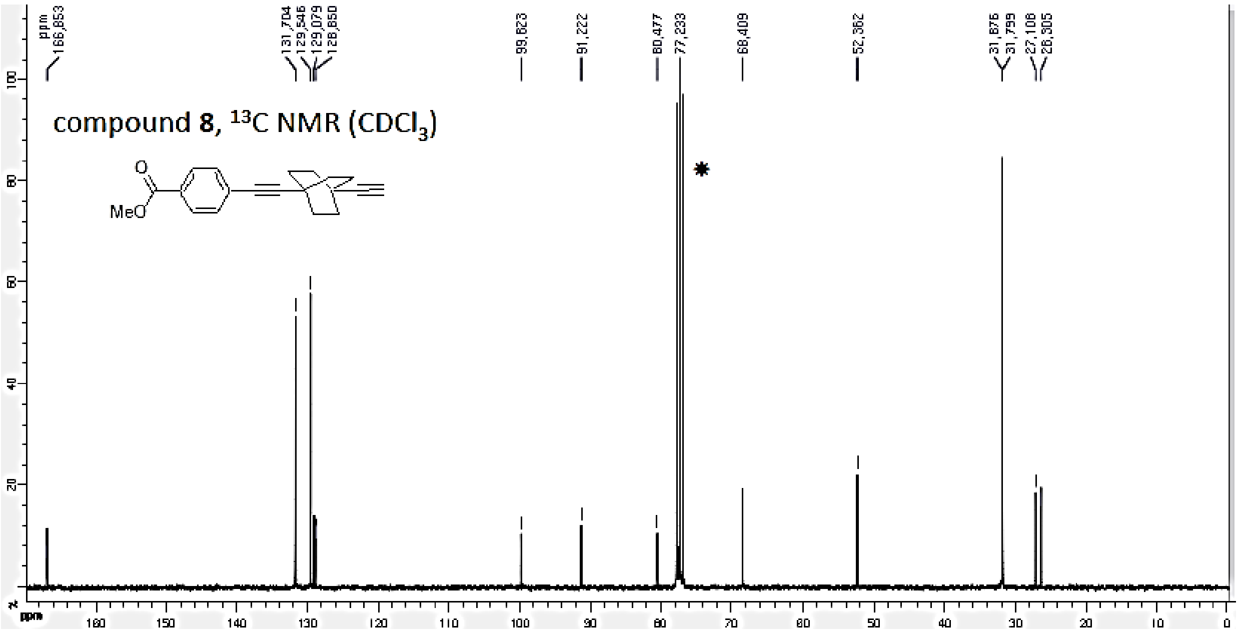

Figure S10. ^{1}H and ^{13}C NMR of compound **8** at 300 MHz. On each spectrum the solvent peak is labeled with a star.

Bruker Daltonics DataAnalysis 3.3 | Page 1 of 1

Display analysis

Sample Name **CL717-1**

Analysis Info	Acquisition Date 4/8/2011 3:35:37 PM	Operator	Ingrid
		Instrument	esquire3000 plus

Analysis Name D:\Data\Ingrid\08042011-CL-717-1.d

Method tune esi.m

Sample Name CL717-1

Comment

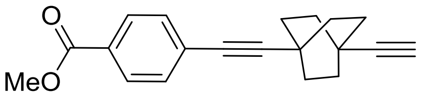

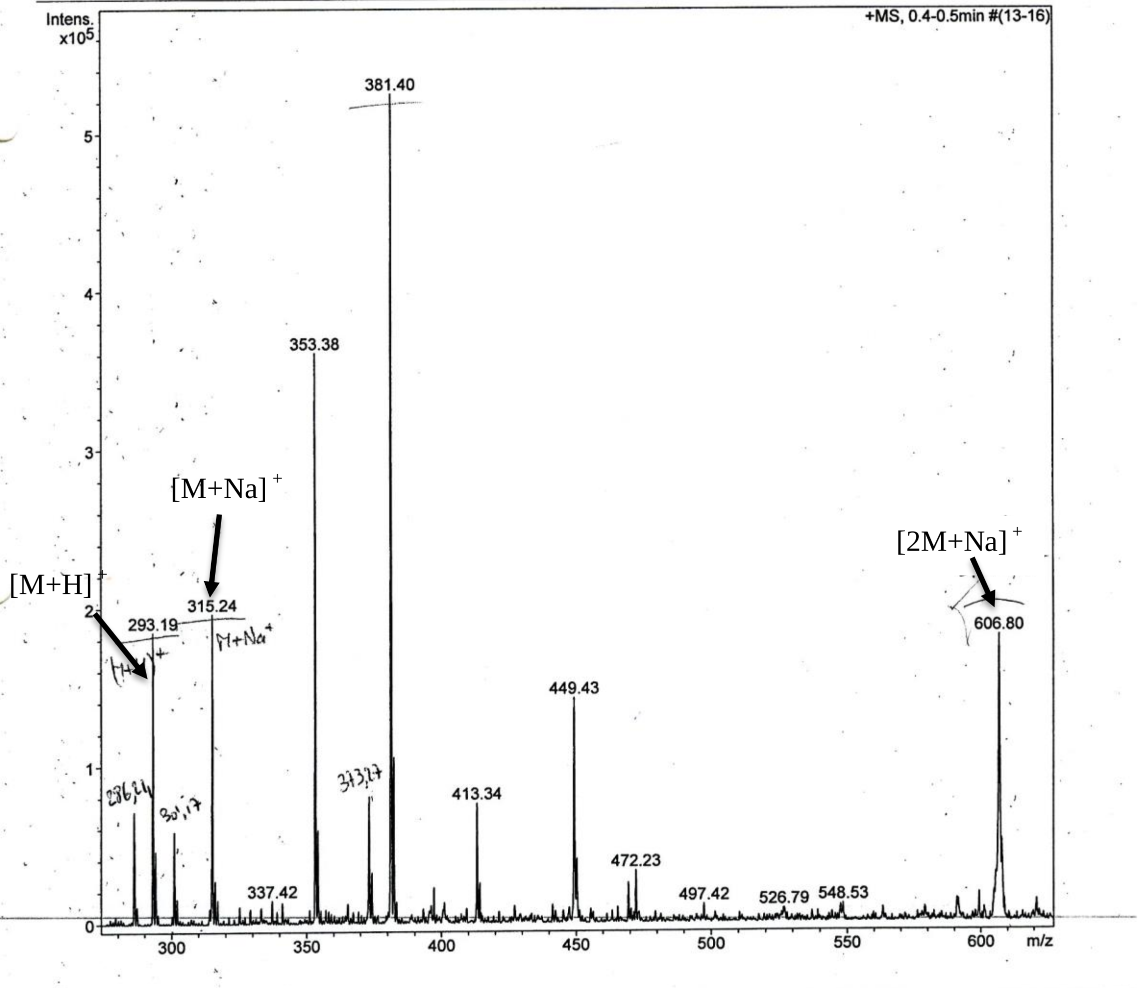

Plateforme d'Ingenierie et Analyses Moleculaires PIAM
Universite d'Angers 2 bd Lavoisier 49 045 Angers cedex 01
Laboratoire de spectrometrie de masse
Tel : 02.41.73.50.87 Fax : 02.41.73.50.07 Email : sylvie.fournier@univ-angers.fr

université angers

PIAM

Figure S11. Mass spectra of compound **8**

1.8 Synthesis and characterization of dimethyl 4,4'-(4,4'-(buta-1,3-diyne-1,4-diyl)bis(bicyclo[2.2.2]oct-1,4-diyl))bis(ethyne-2,1-diyl)dibenzoate (9)

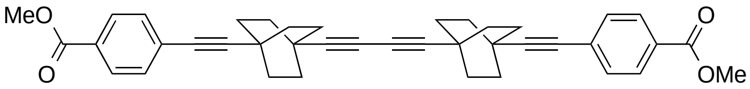

Starting from **8** (100 mg, 0.342 mmol), and following the procedure applied to obtain **6** from **5**, the reaction progress was monitored by thin layer chromatography, (ethyl acetate/*n*-heptane = 50: 50). The product was purified by column chromatography (ethyl acetate/*n*-heptane 50:50) to afford 70 mg (70% isolated yield) of **9** as a whitish solid that is crystallized upon slow evaporation of a solution of **9** (ethyl acetate/*n*-heptane = 50: 50), to yield colorless thin plate-shaped crystals. ^{1}H NMR (300 MHz, $CDCl_3$): δ 7.9 (d, 4H, *J* = 9 Hz), 7.4 (d, 4H, *J* = 9 Hz), 3.9 (s, 6H), 1.8 ($s_{(broad)}$, 24H). ESI(+) m/z = 568.3 $[(M\text{-}CH_3)+H]^+$; 1137,3 $2[(M\text{-}CH_3)+H]^+$.

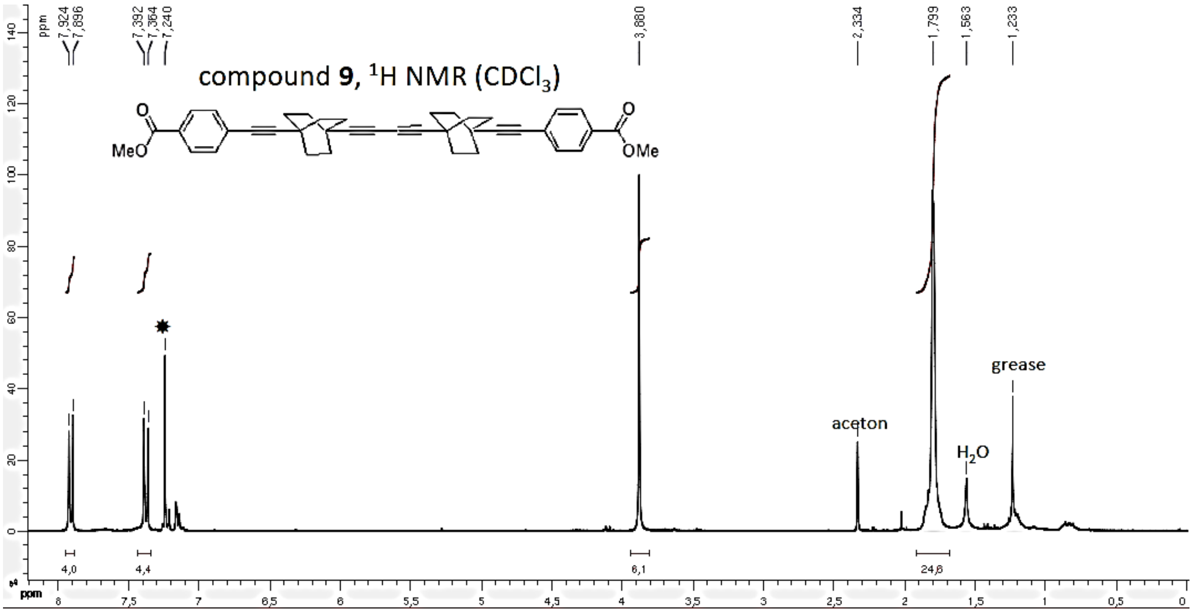

Figure S12. ^{1}H NMR of compound **9** at 300 MHz. The solvent peak is labeled with a star.

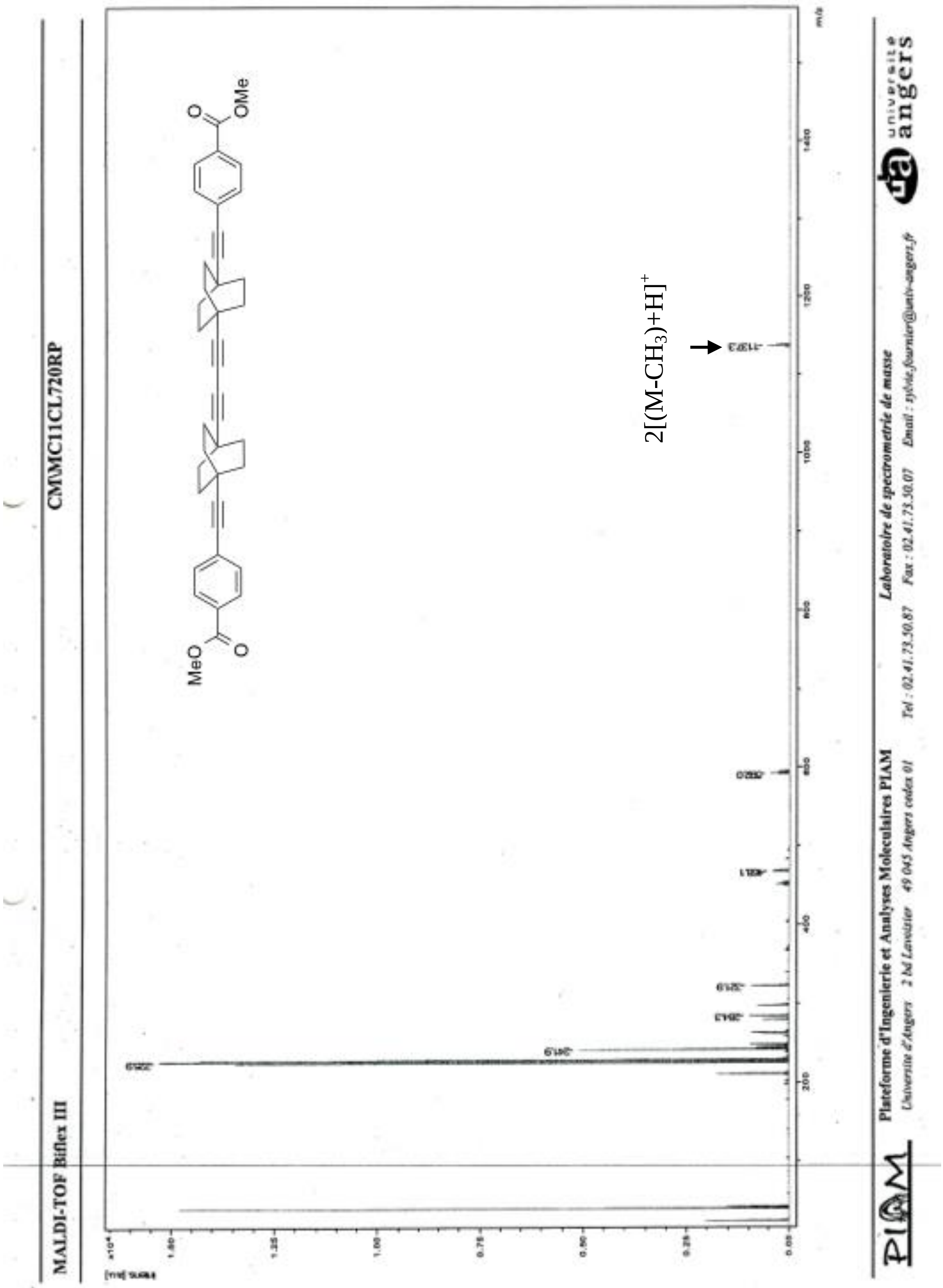

Figure S13. Mass spectra of compound **9**

1.9 Synthesis and characterization of 4-((4-ethynylbicyclo[2.2.2]oct-1-yl)ethynyl)benzoïc acid (11)

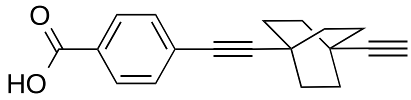

KOH (48 mg, 0.86 mmol) was added to a solution of methyl-4-((4-(3-hydroxy-3-methylbut-1-ynyl)bicyclo[2.2.2]oct-1-yl)ethynyl)benzoate (**7**) (200 mg, 0.57 mmol) in toluene (200 mL) and the mixture was heated at reflux for 2h; the reaction was monitored by TLC (ethyl acetate/*n*-heptan 50:50). The reaction mixture was filtered and the solvent evaporated to dryness under vacuum. The white solid residue was dissolved in THF and washed with an aqueous ammonium chloride saturated solution. The organic layer was evaporated to dryness under vacuum to yield a white solid washed twice with cold water prior overnight drying over P_2O_5 to afford **11** (144 mg, 0.52 mmol) as a white powder in 91% yield. mp dec > 275 °C, ^{1}H NMR (300 MHz, DMSO-d_6): δ 7.9 (d, J= 9 Hz, 2H), 7.4 (d, J= 9 Hz, 2H), 2.9 (s, 1H), 1.8 (m, 12H). ^{13}C NMR (300MHz, DMSO-d_6) δ 131.31, 129.38, 111.07, 99.53, 90.76, 80.00, 71.23, 31.14, 31.06, 26.44, 25.55. ESI(-) m/z = 277.2 [M-H]$^-$, 554.8 . [2M-H]$^-$.

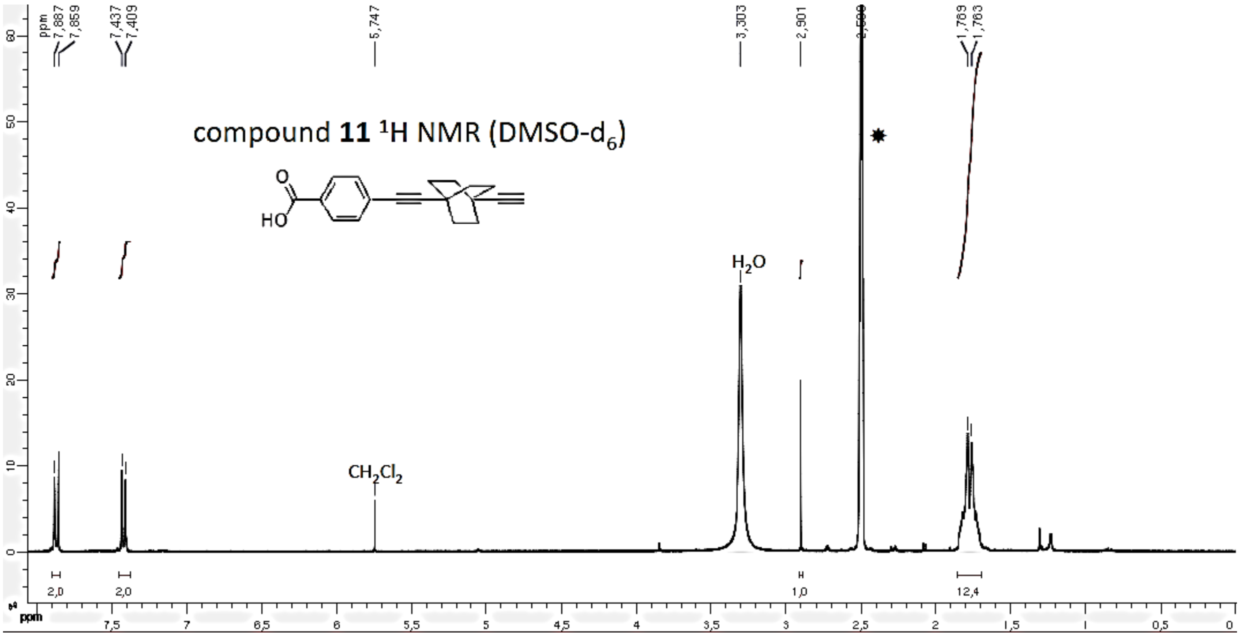

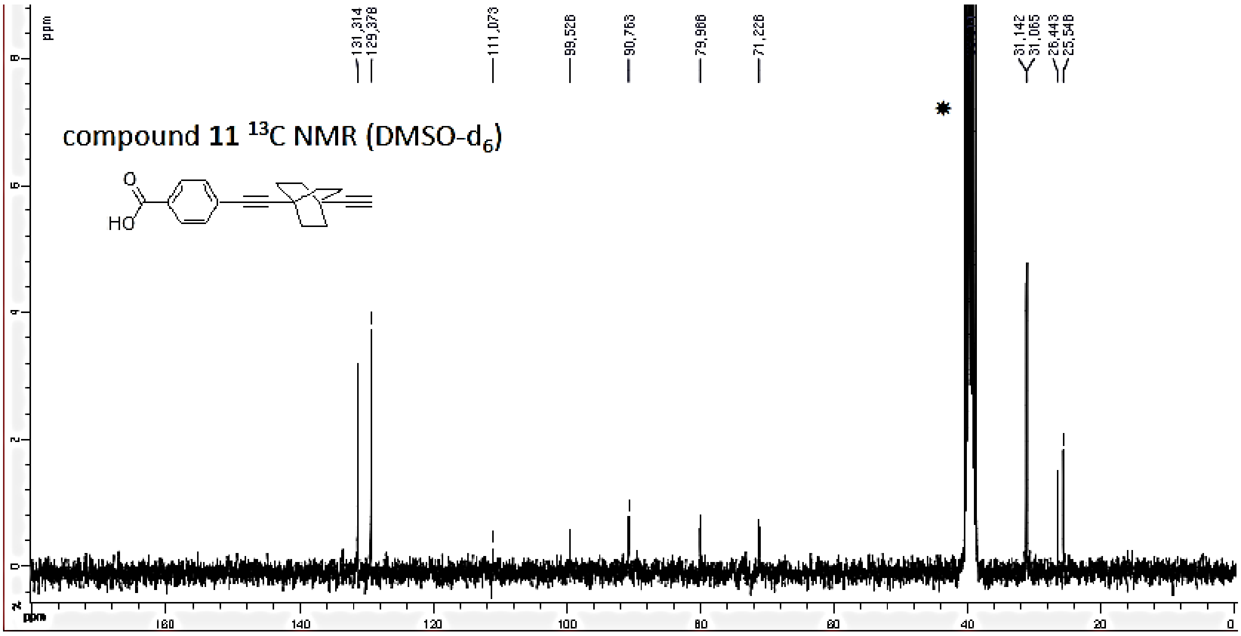

Figure S14. ^{1}H and ^{13}C NMR of compound **11** at 300 MHz. On each spectrum the solvent peak is labeled with a star.

Bruker Daltonics DataAnalysis 3.3 Page 1 of 1

Display analysis

Sample Name CL717-2

**Analysis Info**	Acquisition Date	4/8/2011 2:48:43 PM	Operator	Ingrid
			Instrument	esquire3000 plus

Analysis Name D:\Data\Ingrid\08042011-CL-717-2.d
Method tune esi.m
Sample Name CL717-2
Comment

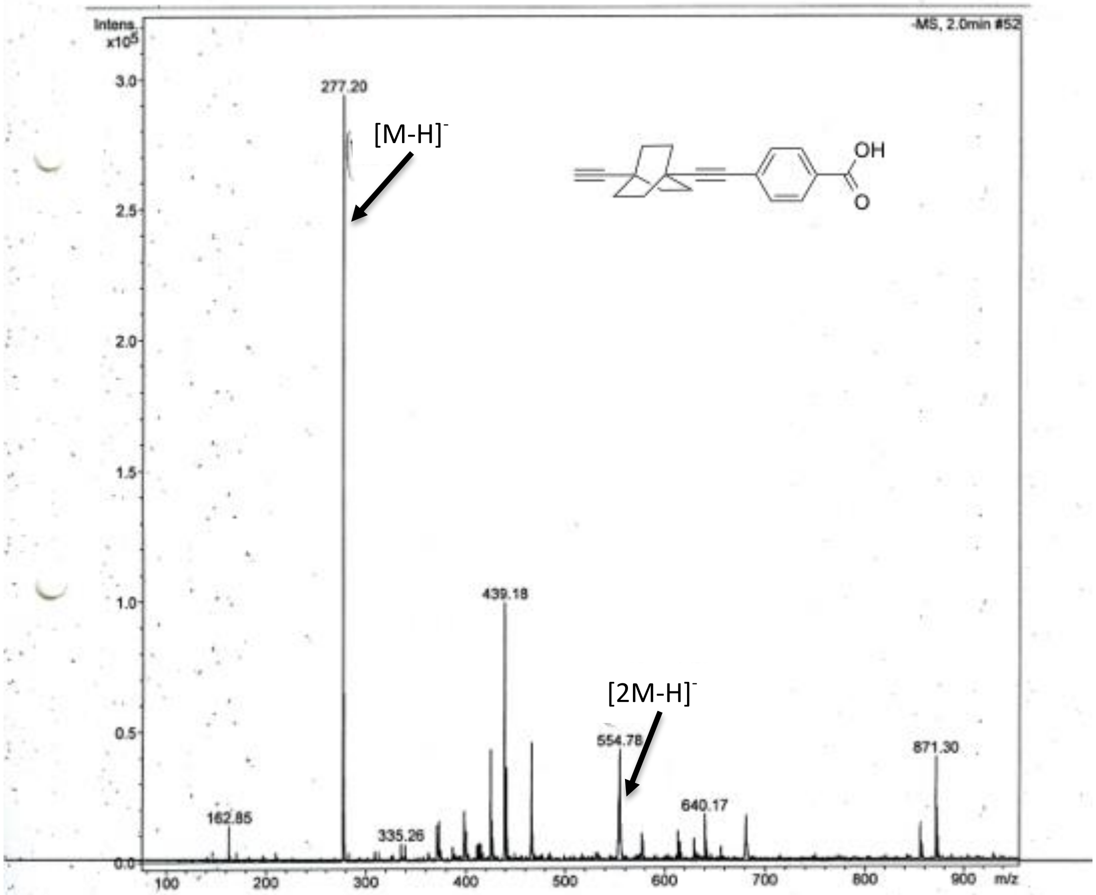

Plateforme d'Ingenierie et Analyses Moleculaires PIAM
Université d'Angers 2 bd Lavoisier 49 045 Angers cedex 01
Laboratoire de spectrometrie de masse
Tel : 02.41.73.50.87 Fax : 02.41.73.50.07 Email : sylvie.fournier@univ-angers.fr

université angers PIAM

Figure S15. Mass spectra of compound **11**

1.10 Synthesis and characterization of 4-((4-ethynylbicyclo[2.2.2]oct-1-yl)ethynyl)-N,N-dimethylbenzamide (12)

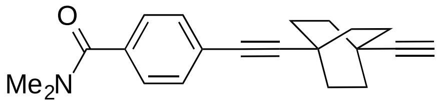

Thionyl chloride (0.4 mL, 5.48 mmol) was added to an homogenous solution of 4-((4-ethynylbicyclo[2.2.2]oct-1-yl)ethynyl)benzoic acid (**11**) (120 mg, 0.43 mmol) in dichloromethylene (10 mL) and the heterogeneous mixture was stirred at room temperature for 2 h, evaporated to dryness and concentrated twice from toluene to yield a white powder which is directly engaged in the second step. Then, under anhydrous and inert conditions, a freshly prepared 20 mL THF solution of acyl chloride was added dropwise to a solution of *N*,*N*-dimethylamine 2 M (10 mL, 20 mmol) provoking instant flocculation. The heterogeneous mixture was stirred at room temperature for 2 h. The reaction was monitored by TLC (ethyl acetate/n-heptane 50:50) and the mixture evaporated to dryness under vacuum to afford a white solid residue purified by column chromatography (ethyl acetate/*n*-heptane 50:50) to yield 98 mg (75% isolated yield) of **12** as a white crystalline powder crystallized by slow evaporation in methylene chloride as colorless plate-shaped crystals. Mp: 189 °C, ^{1}H NMR (300 MHz, $CDCl_3$): δ 7.4 (d, 9 Hz, 2H), 7.3 (d, J= 9Hz, 2H), 3.1 ($s_{(broad)}$, 3H), 2.9 ($s_{(broad)}$, 3H), 2.1 (s, 1H), 1.8 (m, 12H) . ^{13}C NMR (300MHz, $CDCl_3$) δ 171.30, 135.38, 131.67, 127.24, 125.41, 97.99, 91.28, 80.36, 68.36, 39.72, 35.62, 31.90, 31.85, 27.00, 26.30. ESI(+) m/z = 306.31 $[M+H]^+$.

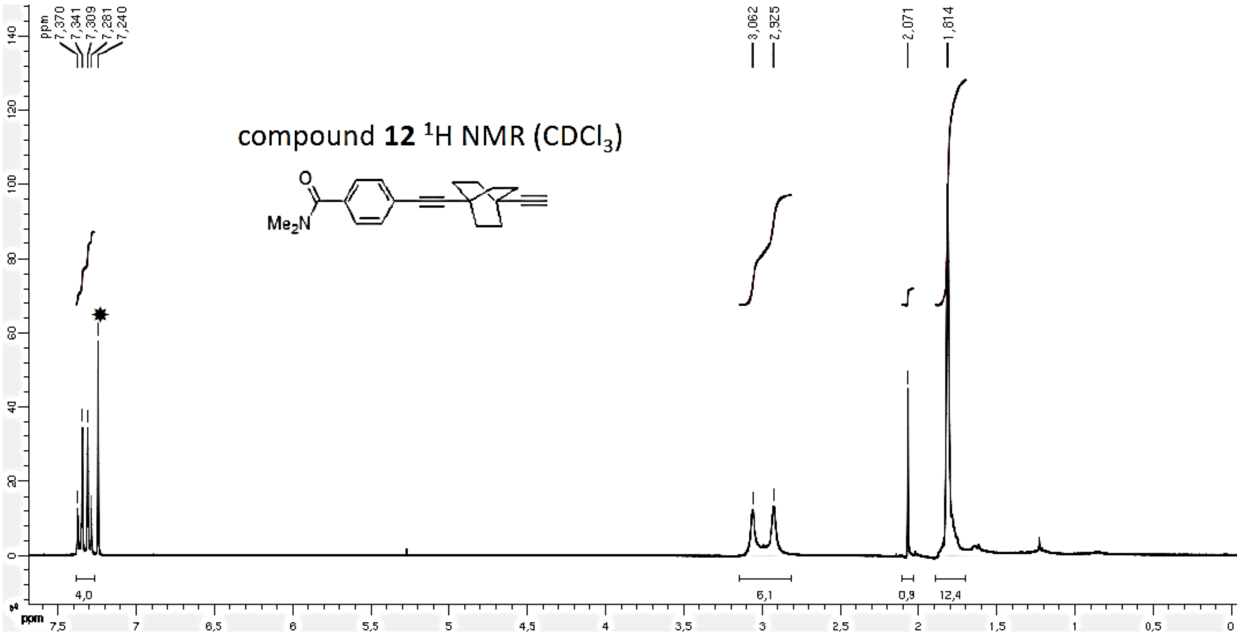

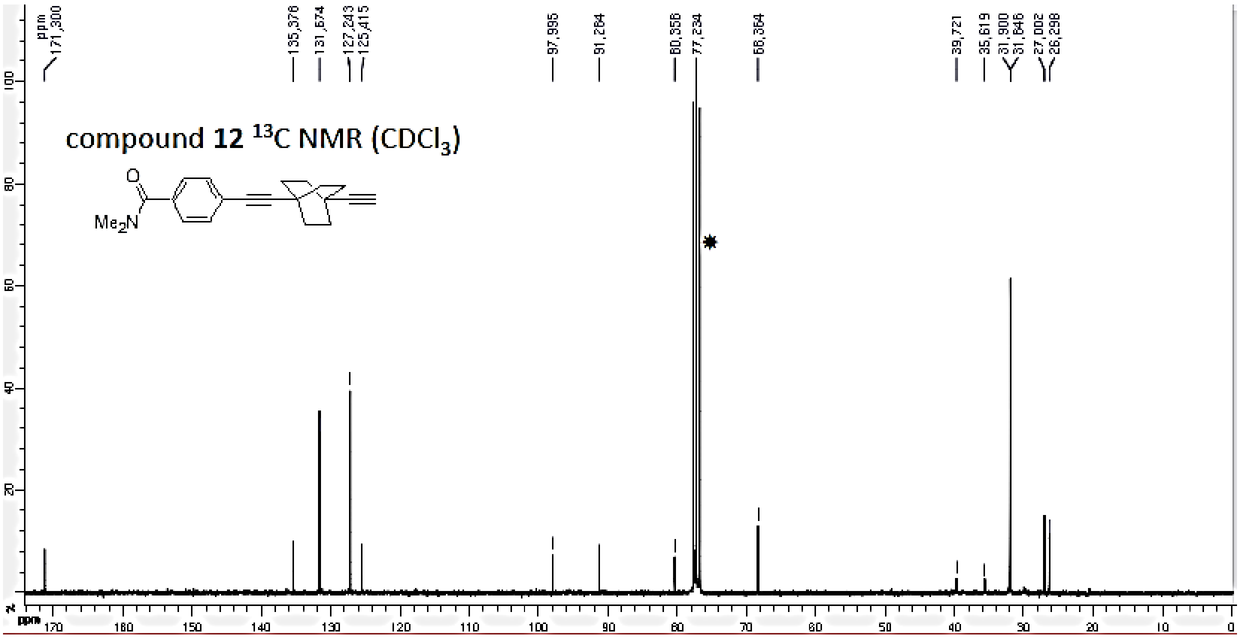

Figure S16. ^{1}H and ^{13}C NMR of compound **12** at 300 MHz. On each spectrum the solvent peak is labeled with a star.

Bruker Daltonics DataAnalysis 3.3 | Page 1 of 1

Display analysis

Sample Name CL759

**Analysis Info**	Acquisition Date 5/10/2011 2:59:05 PM	Operator	Ingrid
		Instrument	esquire3000 plus

Analysis Name D:\Data\Ingrid\10052011-CL-759.d
Method tune esi.m
Sample Name CL759
Comment [illegible]

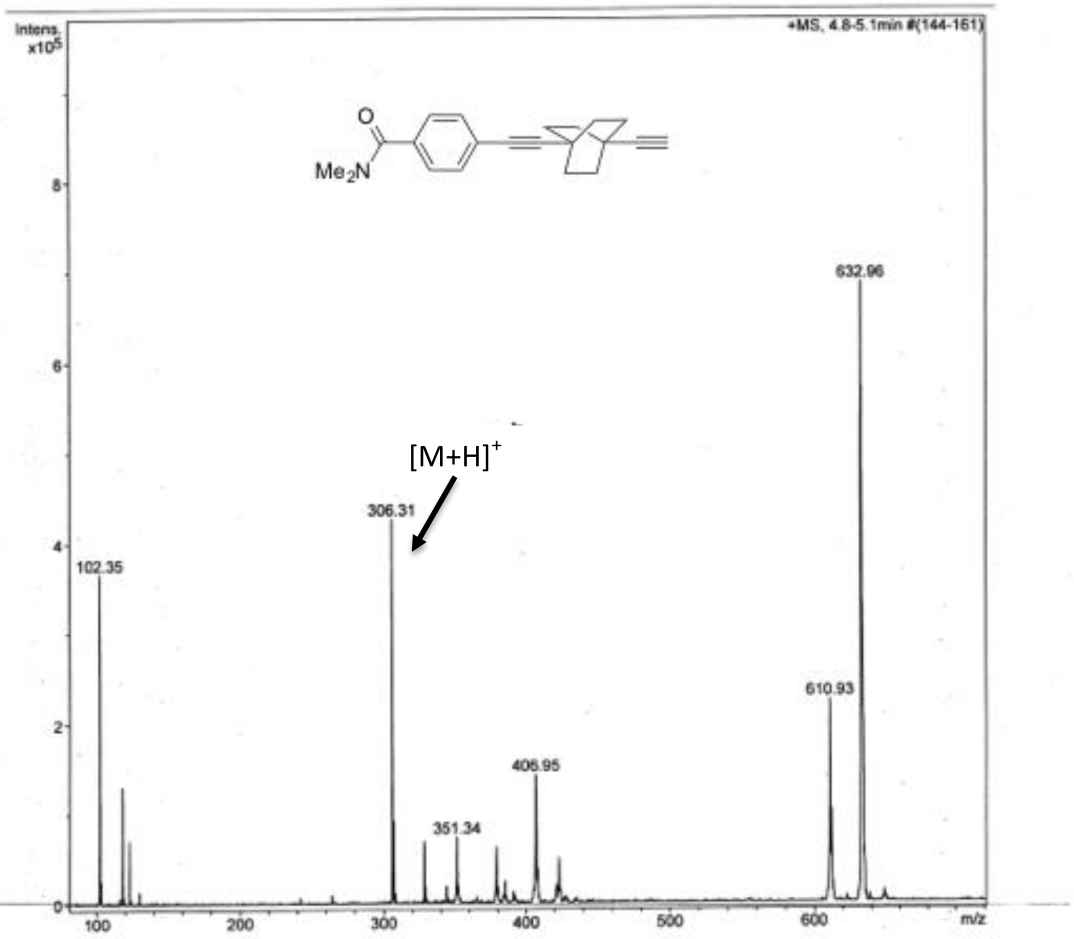

Plateforme d'Ingenierie et Analyses Moleculaires PIAM
Université d'Angers 2 bd Lavoisier 49 045 Angers cedex 01
Laboratoire de spectrométrie de masse
Tel : 02.41.73.50.87 Fax : 02.41.73.50.07 Email : sylvie.fournier@univ-angers.fr

université angers

PIAM

Figure S17. Mass spectra of compound **12**

1.11 Synthesis and characterization of methyl 4-((4-(pyridin-4-ylethynyl)bicyclo[2.2.2]oct-1-yl)ethynyl)benzoate (13)

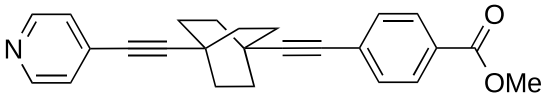

To a solid mixture of 4-iodobenzoate (468 mg, 1.79 mmol), catalytic amounts (ca. 16 mol %) of [Pd(PPh_3)$_4$] and CuI (15 mol %), a solution of 4-((4-ethynylbicyclo[2.2.2]oct-1-yl)ethynyl)pyridine (**5**) (210 mg, 0,89 mmol) dissolved in triethylamine (150 mL) was sparged with argon for 15 min and finally added. The reaction mixture was heated at 70 °C overnight under argon. Reaction progress was monitored by thin layer chromatography (ethyl acetate/*n*-heptane 50:50). The reaction mixture was poured into a mixture of ammonium chloride saturated aqueous solution and methylene chloride (50 mL/50mL) and then extracted. The organics were dried over magnesium sulfate, filtrated and the solvent was removed under vacuum. The product was purified by column chromatography with ethyl acetate/*n*-heptane 50:50 to afford 260 mg (79% isolated yield) of **13** as a yellowhish powder. mp: 173 °C; ^{1}H NMR ($CDCl_3$, 300 MHz) δ 8.5 (d, J = 6 Hz, 2H), 7.9 (d, J = 9 Hz, 2H), 7.4 (d, J = 9 Hz, 2H), 7.3 (m, 2H) *overlap of the signal with solvent peak*, 3.9 (s, 3H), 1.9 ($s_{(broad)}$, 12H) . ^{13}C NMR ($CDCl_3$, 300 MHz) δ 166.85, 149.10, 133.07, 131.73, 129.58, 129.17, 128.80, 126.23, 102.60, 99.60, 80.63, 78.81, 52.38, 31.78, 31.70, 27.28, 27.17; (MALDI-TOF) m/z [M+H]$^+$calcd. 370.17, found 370.20 anal. calcd for $C_{25}H_{23}NO_2$ C, 81.27 ; H, 6.27, found: C, 81.54; H, 6.31.

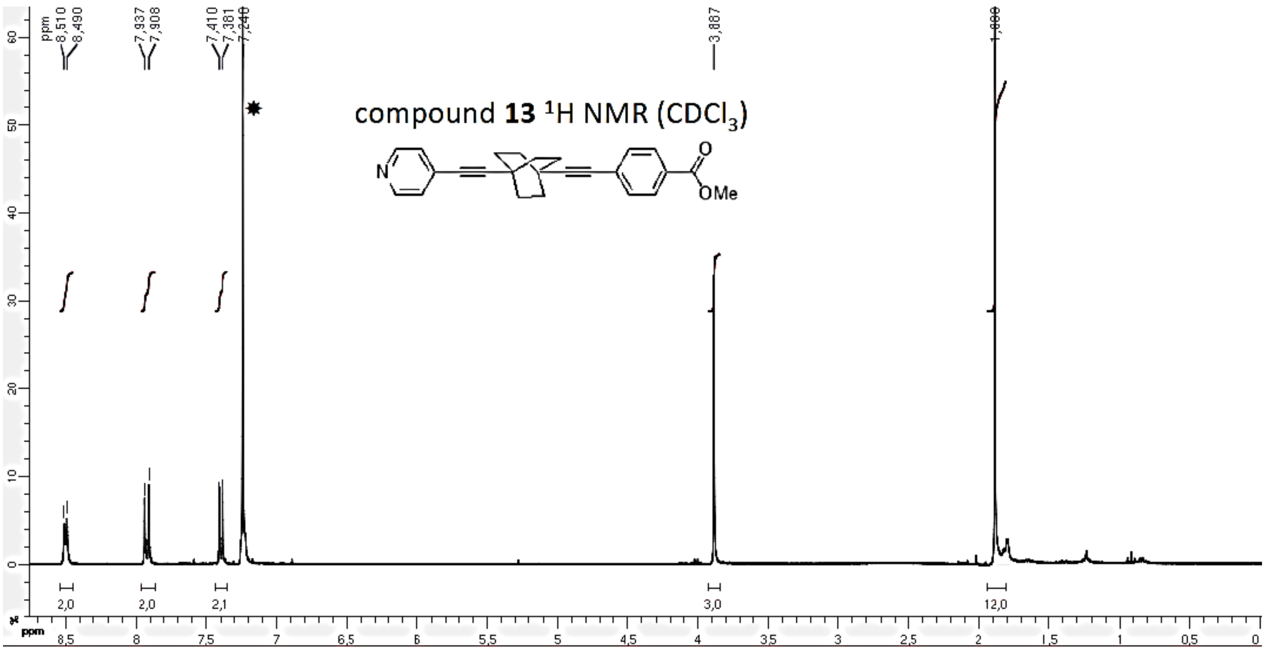

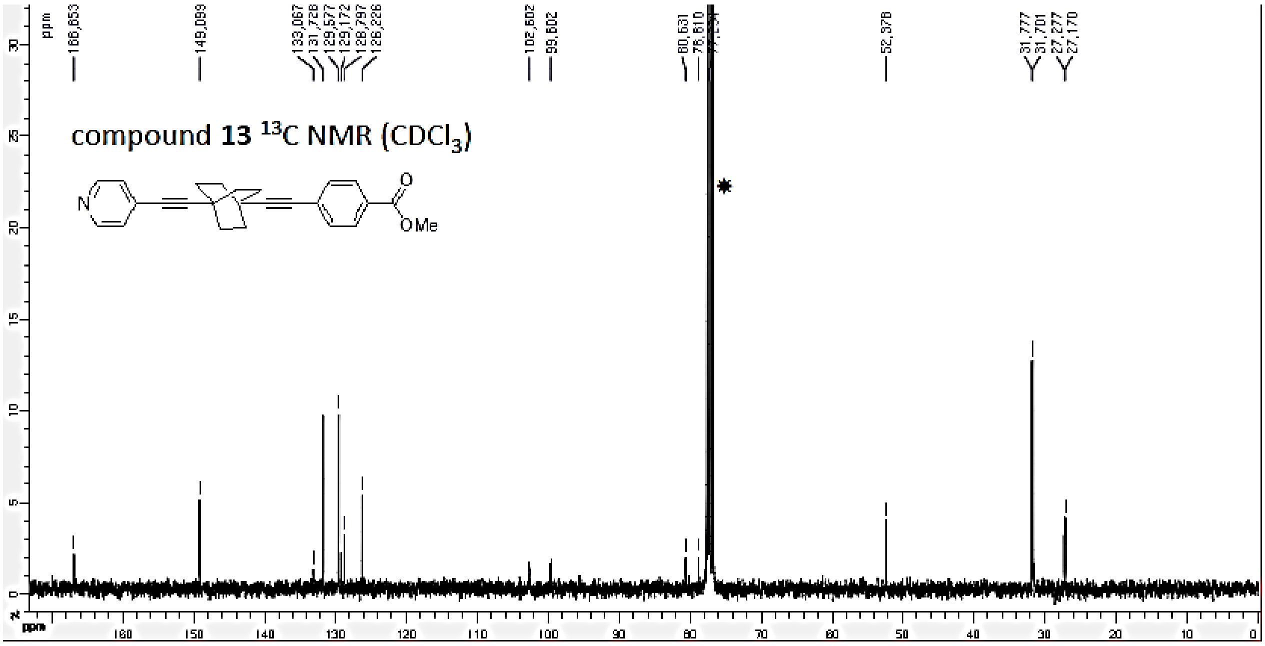

Figure S18. ^{1}H and ^{13}C NMR of compound **13** at 300 MHz. On each spectrum the solvent peak is labeled with a star.

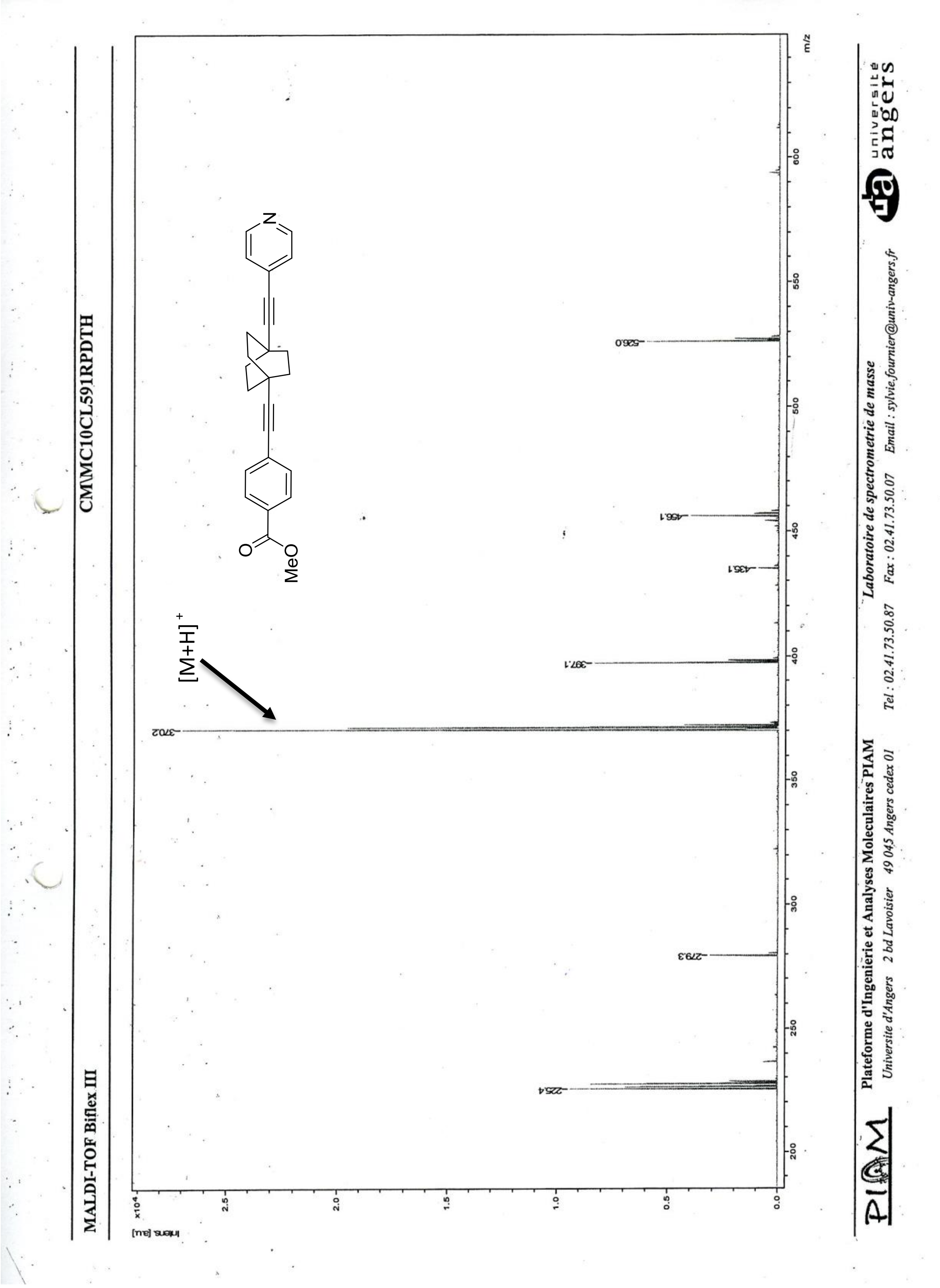

Figure S19: Mass spectra of compound **13**

1.12 Synthesis and characterization of 4-((4-(pyridin-4-ylethynyl)bicyclo[2.2.2]oct-1-yl)ethynyl)benzoic acid (14)

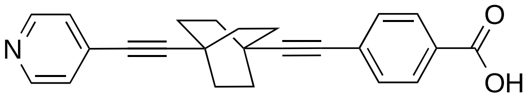

To a solution of 4-((4-(pyridin-4-ylethynyl)bicyclo[2.2.2]oct-1-yl)ethynyl)benzoate (**13**) (200 mg, 0.54 mmol) in 1,4-dioxan (50 mL), a solution of lithium hydroxide (340 mg, 8.13 mmol) in 5 mL of water was added. The reaction progress was monitored by thin layer chromatography (ethyl acetate/*n*-heptane 50:50). The reaction mixture was extracted with tetrahydrofurane and saturating the water layer with ammonium choride. The organic layer was evaporated under vacuum and treated three times with toluene to remove water traces and the solid was dried over P_2O_5 for 2 days to afford **14** (174 mg, 0,49 mmol) as a white powder in 91% yield. mp. dec. > 400°C ; ^{1}H NMR (DMSO-d_6, 300 MHz) δ 8.5 (m, 2H), 7.9 (m, 2H), 7.4 (m, 2H), 7.3 (m, 2H), 1.9 (m, 12H); ^{13}C NMR (DMSO-d_6, 300 MHz) δ 149.80, 131.31, 129.40, 125.51, 124.51, 31.04, 30.93, 26.57, 26.50; ESI-HRMS(-) m/z [M] calcd 355.160 , found 355.153 ; [M-H]$^-$calcd 354.150, found 354.150.

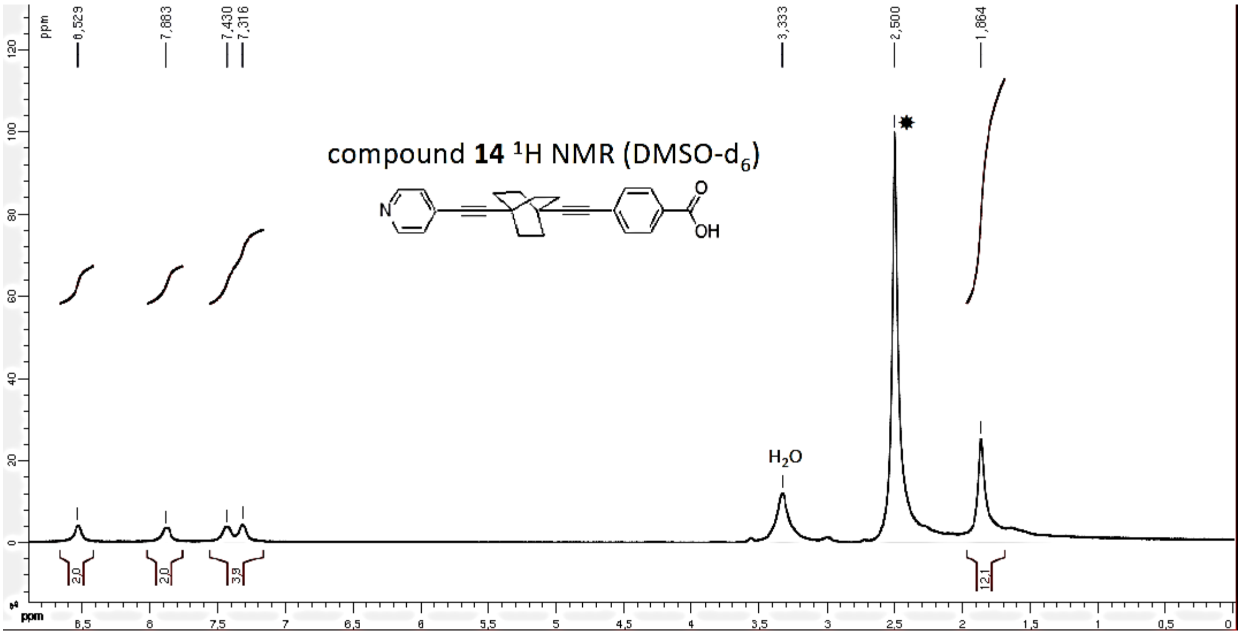

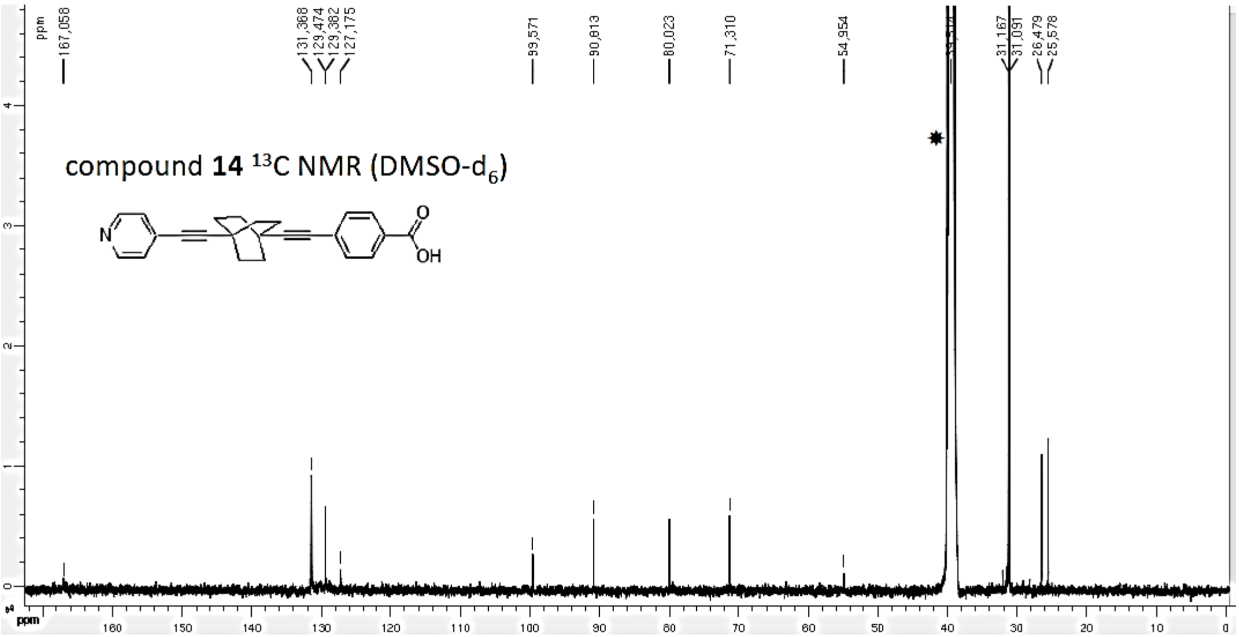

Figure S20. ^{1}H and ^{13}C NMR of compound **14** at 300 MHz. On each spectrum the solvent peak is labeled with a star.

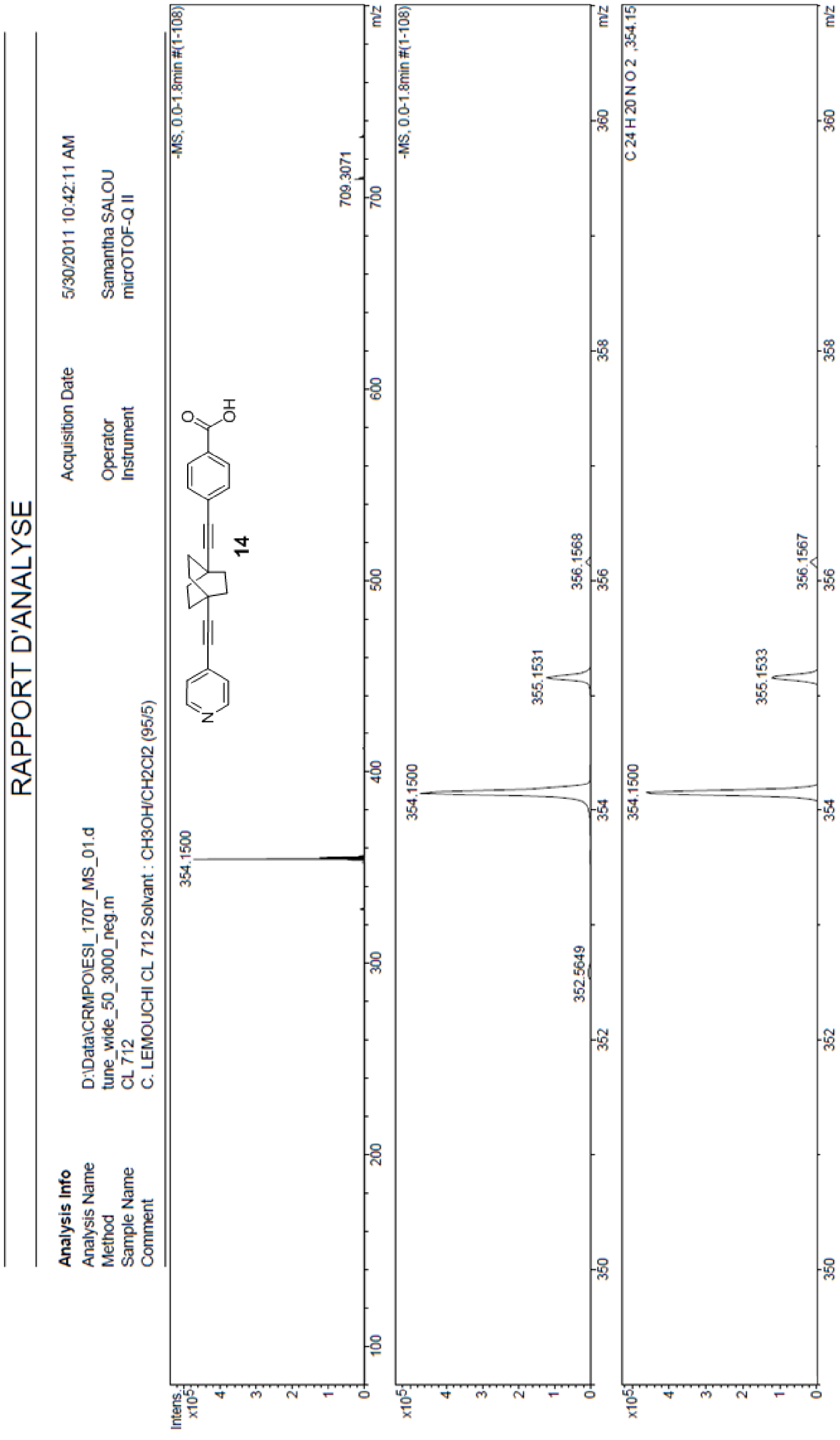

Figure S21. High Resolution Mass spectra of compound **14**
